# An integrated approach to epitope analysis II: A system for proteomic-scale prediction of immunological characteristics

**DOI:** 10.1186/1745-7580-6-8

**Published:** 2010-11-02

**Authors:** Robert D Bremel, E Jane Homan

**Affiliations:** 1 1ioGenetics LLC, 3591 Anderson Street, Madison, WI 53704, USA

## Abstract

**Background:**

Improving our understanding of the immune response is fundamental to developing strategies to combat a wide range of diseases. We describe an integrated epitope analysis system which is based on principal component analysis of sequences of amino acids, using a multilayer perceptron neural net to conduct QSAR regression predictions for peptide binding affinities to 35 MHC-I and 14 MHC-II alleles.

**Results:**

The approach described allows rapid processing of single proteins, entire proteomes or subsets thereof, as well as multiple strains of the same organism. It enables consideration of the interface of diversity of both microorganisms and of host immunogenetics. Patterns of binding affinity are linked to topological features, such as extracellular or intramembrane location, and integrated into a graphical display which facilitates conceptual understanding of the interplay of B-cell and T-cell mediated immunity.

Patterns which emerge from application of this approach include the correlations between peptides showing high affinity binding to MHC-I and to MHC-II, and also with predicted B-cell epitopes. These are characterized as coincident epitope groups (CEGs). Also evident are long range patterns across proteins which identify regions of high affinity binding for a permuted population of diverse and heterozygous HLA alleles, as well as subtle differences in reactions with MHCs of individual HLA alleles, which may be important in disease susceptibility, and in vaccine and clinical trial design. Comparisons are shown of predicted epitope mapping derived from application of the QSAR approach with experimentally derived epitope maps from a diverse multi-species dataset, from *Staphylococcus aureus*, and from vaccinia virus.

**Conclusions:**

A desktop application with interactive graphic capability is shown to be a useful platform for development of prediction and visualization tools for epitope mapping at scales ranging from individual proteins to proteomes from multiple strains of an organism. The possible functional implications of the patterns of peptide epitopes observed are discussed, including their implications for B-cell and T-cell cooperation and cross presentation.

## Background

The availability of proteomic information is increasing exponentially. This is especially true for pathogenic microorganisms. Integration and interpretation of vast amounts of data from the analysis of proteomic information, so that it may be useful to bench scientists and clinicians is a growing challenge. Achieving this goal is essential if bioinformatic analysis is to lead to improved vaccines and antibody therapies and to a better understanding of patient and population responses to infections, cancers, autoimmune epitopes, and allergens. Experimental approaches to definition of epitopes are time consuming and expensive; predictive methods can provide maps which could reduce the effort needed in experimental characterization.

### Current Challenges in Epitope Analysis

In reviewing approaches to epitope characterization described in the literature, both experimentally and through the use of computer-based analysis, three broad shortcomings become apparent.

First, literature reports of experimental approaches to epitope characterization have often been narrow in scope, based on the response of individual patients, cells from a few individual donors or single strains of mice, or focused on isolated peptides. This has generated valid data, but which is specific to the narrow set of circumstances and not reflective of the broader host or organism population. Discovering binding affinity for an MHC molecule of a single HLA haplotype will not necessarily be predictive for a population of diverse heterozygotic individuals. Many literature reports claim T-cell epitope characterization but fail to report the MHC restriction (mouse) or HLA of cells used. By limiting consideration to isolated peptides, an important feature of cell biology is overlooked. Binding to MHC-I and MHC-II molecules is a competitive and dynamic process [1,2]. MHC molecules bind to peptides selected from among all those competitors which result from the proteolysis of the whole organism. Predictive determinations of preferential epitope binding can thus only be made when considered in the context of the whole proteome, or, at very least, the whole protein, but not for isolated peptides.

Second, from an epidemiologic perspective the outcome of infection is dependent on the interface between a population of heterozygous hosts and a diverse array of microbial strains. Many possible interactions of individual and strain are possible. Depending on the context, the challenge in vaccine design may be to choose the best combination of epitopes conserved across multiple strains of an organism to protect an entire immunogenetically diverse community (for infectious diseases), or to select the immunostimulant optimal for a specific patient (in cancer immunotherapeutics).

Third, while there is broad recognition that strong T-cell responses are essential to good memory, and in many cases to effective immunity, efforts to characterize B-cell and T-cell responses have not always been well integrated.

B-cell and T-cell cooperative interaction in antigen presentation has been the subject of many landmark papers [3-6]. More recently, Sette *et al *demonstrated that, at least for vaccinia, T-cell stimulation is specific to a B-cell epitope located within the same protein [7], pointing to a close determinant association between B-cell and T-cell epitopes. Cross reactivity, or polyspecificity, is a necessary feature of the T-cell recognition of epitopes comprised of MHC-peptide complexes [8,9].

There has been increasing recognition that, both for anti-infective immunity, and for cancer immunity, distinctions between the role of MHC-I and MHC-II in responding to intra or extra-cellular organisms are not clear cut [10-13]. MHC-II molecules bind longer peptides (15-20 amino acids) whereas MHC-I molecules bind shorter peptides of ~9 amino acids or less [1]. Binding of MHC molecules to peptides is characterized by a large degree of degeneracy and it is now recognized that a particular MHC molecule may bind peptides that vary widely in composition and origin [9].

B-cell epitopes may be continuous or discontinuous peptides, in some cases requiring multiple linear peptides to be configured together to make up a complete epitope [14]. Location of B-cell epitope motifs in loops external to the cell membrane may allow for grouping into a multi-component epitope. Multiple peptides may need to act together to provide an immunostimulant adequate to initiate a B-cell response. Batista has described the need for B-cells to have sufficient stimulation to form immune synapses, initiating and enabling the uptake of surface proteins [15]. In other cases B-cell responses occur independent of T cell stimulation [16].

Most successful antimicrobial vaccines target surface exposed B-cell epitopes and vaccines have been evaluated by their ability to stimulate an antibody response. Peptide epitopes are a major component of the overall epitope complex, or immunome, and are genetically specified. In many cases antibodies to bacterial proteins are indeed protective, and complement fixing antibodies have been used as an index of vaccinal efficacy [17].

Immunization protocols for laboratory production of antibodies have long recognized the utility to linkage to a known T-cell epitope [18,19]. T-cell responses to epitopes arrayed in an organism of interest are harder to evaluate [20]. Those working in reverse vaccinology [21] have been frustrated by the difficulty of reliably characterizing T-cell epitopes [17]. Proteins with multiple transmembrane domains have proven challenging to express as sub-unit vaccines [21]. In understanding the interaction of B-cell and T-cell responses, it is therefore useful to readily understand the topology of epitopes relative to the cell membrane. In the case of immunotherapeutic cancer vaccines, the ability to stimulate a multifaceted T-cell response may be even more necessary [22,23].

### State of the Art: Epitope prediction programs

Various bioinformatic programs for B-cell epitope and T-cell epitope analysis are available on the Internet (Additional File 1) and have contributed significantly to our understanding. However, a number of limitations are evident. Limits on the sequence size which can be submitted to website servers generally only allows single protein analysis and thus preclude contextual understanding of competitive binding affinity for a whole proteome.

#### B-cell epitope predictions

Schemes for prediction of B-cell epitopes have been available for nearly 30 years. Hopp and Woods [24] first proposed the use of amino acid sequences to identify the most immunogenic regions in proteins and recognized the relative importance of surface exposure, a concept furthered by Parker *et al *[25]. By using various lengths of peptides as indices to produce scoring metrics, about 70% of the epitopes in a small set of proteins could be accurately predicted. A wide array of methods has been published since, but the predictive performance has not greatly improved [26]. The field has recently been critically reviewed by Davydov and Tenevitsky [27], who use a preferred binary classification metric AROC method of evaluation. Recalculated, the accuracy reported by Hopp and Wood and the contemporary AROC values are not substantially different.

The availability of the BepiPred program over the Internet (on the servers at the Center for Biological Sequence Analysis (CBS)), and its ability to process partial proteome-scale sequence data, led us to initially utilize this program [28]. Interestingly, the algorithms rely heavily on the work of Parker [25]. We subsequently found that the amino acid principal components NN regression approach, which we describe in the accompanying paper [29], and which uses the physical property data sets of Hopp and Woods [30], Parker *et al *[25], and others, could produce outputs indistinguishable from BepiPred [28]. This enabled us to consolidate the computations into a single platform along with MHC binding predictions and facilitated integration with genomic data processing programs.

#### MHC binding Predictions

DeGroot reviews T-cell epitope mapping systems available publically and developed commercially [31]. Many T-cell epitope prediction programs depend on substitution matrix scoring of individual amino acids. As we have discussed in a companion paper [29], this does not provide a complete physicochemical description of the binding relationship. Substitution matrices are the backbone of bioinformatics, but were originally developed to assist in understanding evolutionary genetic relationships, not physicochemical properties. Quantitative structure activity relationship (QSAR) approaches that utilize the physicochemical properties of interacting species as a foundation are a more appropriate method. These have been applied by one group but in the context of peptides rather than proteins or proteomes [32-36].

The bioinformatic approaches currently available and discussed above are designed to analyze B-cell epitopes or T-cell epitopes but, despite the recognized interplay of B and T cells, fail to integrate the two to provide a complete picture of the immunome.

Virtually all website based programs understandably place limits on sequence size. Further complicating this is the absence of uniformity in size limitations, making consistent data manipulation challenging. The outputs are difficult to integrate when obtained piecemeal. More importantly, from a practical viewpoint, software reliability testing over the internet is at best challenging. Where the programs can be acquired for local use, the Unix/Linux platforms favored by the bioinformatics community are not commonly available in laboratory settings so converting the programs into functional utility in a local setting is not trivial.

Our first goal was thus to produce a unified system, that consolidated the various immunological metrics into one set of tools and operated within the context of commercially available software on widely-used computing platforms. MHC-I and MHC-II binding using the neural network and partial least squared platforms of JMP^® ^(also JMP^® ^Genomics) http://www.jmp.com is described in an accompanying paper [29]. Secondly, we recognized the need to examine the interface of immunogenetically diverse patient populations along with an array of different strains of the same organism. Thirdly, we considered a graphical display that allowed visualization of the output of very complex statistical computations to be desirable. Our conceptual model in approaching this third goal was the superior level of understanding of land use provided by geographic information systems (GIS) which overlay multiple information sets of physical and economic geography. We have applied this concept to the microbial surfome "landscape". In this paper we describe an integrated bioinformatics analysis system which we believe approaches these goals.

## Methods

### Selection of Benchmark Datasets

We sought appropriate benchmark datasets to test the system developed. None were available which provided comparable levels of information on all the features we sought to integrate. While a useful repository, the IEDB tabulation of individual epitopes is less useful for proteomic-scale work. We selected three datasets for comparison. The "AntiJen" database provided a benchmark for evaluating a diverse repository of epitopes within the context of entire protein molecules. Two well studied infectious organisms, *Staphylococcus aureus *and vaccinia virus, enabled retrospective comparisons with published data.

#### AntiJen

We examined reference datasets of mapped B-cell epitopes on various websites. Additions or subtractions of sequences have been made to some datasets (reviewed in Davydov [27]). We sought datasets where epitopes had been mapped for the entire length of a protein and which provided a wide array of source proteins. We downloaded the datasets of identified B-cell epitopes from the site at CBS. The largest one, labeled "AntiJen", is a derivative of that described by Toseland *et al *[37] (but no longer available at the weblink provided in this publication). From the annotations, some of the proteins appear to trace to the time of Hopp and Woods [24]. This dataset may be accessible from other websites but we report herein our use of it as downloaded from CBS [38,39] (currently accessible at http://www.cbs.dtu.dk/suppl/immunology/Bepipred.php).

As downloaded, the "AntiJen" data set comprised 124 proteins spanning mammalian, viral, protozoan, bacterial, and other origins (Additional File 2, Table S2a), in which 246 B-cell epitopes have been defined experimentally by various labs and various methods. Larsen *et al *state that "the proteins of this data set are not fully annotated, and the annotation for the non-epitope stretches is not known" [28].

#### Staphylococcus aureus

Many experimental studies have been conducted to define epitopes on several proteins of *Staph. aureus *(See Additional File 3, Table S3a). Proteomes of multiple strains are available in Genbank. We worked with the 15 strains listed in Additional File 3, Table S3b.

#### Vaccinia

In view of the detailed experimental epitope mapping information available for vaccinia [11,40-42] and the demonstration by Sette *et al *[7] of the deterministic linkage of B-cell and T-cell epitopes at a protein level in the I1L core protein of vaccinia, we also processed the vaccinia proteome, and report on the results for a subset of four proteins as an illustration of the use of the integrated system to provide predicted HLA-specific differences in binding affinity.

### Process Description

**Figure 1:**
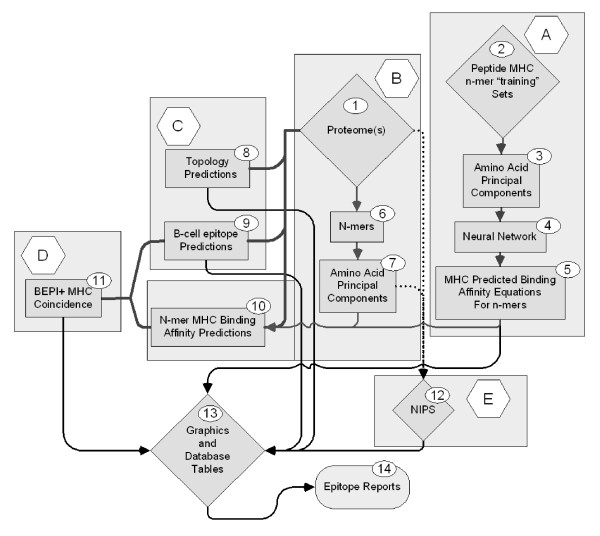
**Elements of peptide epitope prediction process**.

A system for integrated analysis of proteome scale epitope information was designed which comprises a number of sub-processes. All computations were done and graphics generated using JMP^® ^version 8 http://www.jmp.com. Figure 1 provides an overview of the system; the sub-processes are described briefly below and in detail in Additional File 4.

**Process A **consists of developing a set of Neural Network (NN) binding predictions for 14 MHC-II and 35 MHC-I molecules. Once developed these equations are stored for further use. Briefly, principal component amino acid analysis was carried out on the physical properties of amino acids measured in a total of thirty-one different published studies. In the NN each amino acid is assigned 3 numerical values based its principal components rather than the standard alphabetical representation commonly used in bioinformatics. This type of descriptor is commonly used in QSAR analysis where it is known as the "z"-scale [43,44]. The principal component descriptors are uncorrelated, mutually orthogonal metrics, and embody about 90% of the variance in all physical properties of the 20 amino acids commonly found in proteins. The z-scales are not in themselves physical properties, but rather uncorrelated dimensionless proxies for amino acid physical properties that can be used predictively: z_1 _is a hydrophobicity or polarity correlate, z_2 _a size correlate and z_3 _an electronic correlate. A characteristic of principal component analysis is that it also produces a set of descriptors that are appropriately weighted for regression analysis. This process is described in detail in a companion paper where it is benchmarked against several other prediction schemes [29].

**Process B**, also described in the companion paper, consists of replacing the alphabetic notation of amino acids by z-scales so that each 9-mer in the proteome is represented as a vector of 27 numbers and each 15-mer as a vector of 45 numbers. These numerical values are then used to compute predicted binding affinities for peptides in the proteome using the NN prediction equations from Process A.

**Process C **involves the use of one of several publicly available programs for protein topology predictions. We have variously used PHOBIUS [45], PHILIUS [46], MEMSAT [47], and TMH [48]. The output is a probability prediction for each amino acid in the protein as being intracellular, extracellular, within a membrane or a signal peptide. A determination of B-cell epitope predictions is also made. Unlike the MHC predictions which provide a predicted affinity, in the case of B-cell epitopes we are making a binary "yes-no" probability prediction that a specific amino acid lies in a B-cell epitope. The B-cell epitope probability may be achieved by submission to one of several publicly available programs for B-cell epitope predictions [28], however we generated a B-cell epitope prediction based on principal components, enabling us to achieve this step as an integral part of the process.

**Process D **integrates the output from the first three processes and involves the use of self-organizing mapping algorithms to identify Coincident Epitope Groups (CEG) for protein segments likely to be accessible to the immune system. CEGs are peptides in which high affinity MHC binding peptides and B cell epitopes are found to overlap or whose borders lie within a user-specifiable distance of each other. As described herein the distance was set at 3 amino acids.

**Process E **is a database task to assemble nearly identical protein sets (NIPS) from different strains of organisms to arrive at a minimalist set of conserved or near-conserved peptide epitopes for further consideration.

### Standardization

To facilitate further statistical procedures, the MHC binding affinities (as natural logarithms) were standardized. Standardization is a common statistical process where the data points are transformed to a mean of zero and unit variance (and standard deviation as the standard deviation is the square root of the variance). Thus all binding affinities of all different supertypes, and paired supertype combinations, were put on the same basis for further computations. This process is reversible so that a more experimentally meaningful ic_50 _can be obtained at any point if desired. Secondly, the Bayesian probabilities for each individual amino acid being in a B-cell epitope were subjected to global standardization like that for the MHC binding affinities. Thus, all the peptides and other metrics subject to statistical screening are standardized, so that thresholding or other selections are made on single or joint normal probability distributions.

Following the standardization processes, the tables of binding affinities contained columns of the original predicted binding affinity data for the different MHC supertypes (as natural logarithms) and the original B-cell epitope probabilities, as well as corresponding columns of standardized (zero mean, unit standard deviation) data of the immunologically relevant endpoints.

### Design of Graphical Output

Visualization of all epitope components in relation to topology facilitates understanding of function. A graphical scheme (Step 13 in Figure 1) was developed that made it possible to readily visualize the topology of proteins at the surface of the organism as well as three standardized probabilities for high affinity binding petides for MHC-I and MHC-II, and B-epitopes. Predictions for MHC-I and MHC-II binding were done routinely for all organisms, although it is recognized that MHC-I is generally considered most relevant for intracellular infectious organisms and MHC-II for extracellular organisms. Simultaneous visualization of both provides a method of conceptualization of potential cross-presentation of epitopes.

We adopted a convention for graphical display in which the amino acids positions are aligned along the X axis from N to C. The Y axis is in standardized units (zero mean, unit std dev) to show MHC binding affinity. Topological information is displayed in the background shading. The permuted minima ln(ic50) representing the mean population phenotype are plotted for MHC-I and/or MHC-II at each peptide position the number representing a mean of 105 (MHC-II) or 630 (MHC-I) allelic combinations at that position. This is windowed average minima ± 4 amino acids from the plotted point. A smooth line fit through the points is produced using the polynomial filter of Savistsky and Golay [49]. Another line is overlaid to show the standardized probability of B-cell epitope binding. Across the base of the graphic we use ribbons of various colors and intensities to designate regions of high binding affinity and coincidence of B-cell and MHC binding. Different thresholding stringencies can be applied to the ribbons (see Additional File 5); we have mostly found that the 25^th ^percentile of the permuted minimum distribution of MHC binding along with the 25^th ^percentile of B-cell epitope probability to be useful thresholds and these are used throughout the graphics below. The 25^th ^percentile should be clearly understood to be a threshold from within the permuted minima distributions that have their own mean and standard deviations, and not the 25^th ^percentile of all peptides. Colored vertical lines are used to show the behavior of any particular HLA allele as compared to the permuted population phenotype. The line extends from the permuted population value to the standardized value of the indicated HLA at the N-terminus of the peptide 9-mer or 15-mer. By creating a further overlay experimentally defined epitopes can be compared with predictions. The display can easily be rescaled to visualize the individual amino acids in the peptides.

## Results

### Observations of Single Proteomes

Table 1 is summary of the binding affinities for MHC-II supertypes for the surfome (surface proteome) and secretome (secreted proteins) of *Staphylococcus aureus *COL (Genbank genome accession number = NC_002951). The surfome consists of all proteins coded for in the genome that have a molecular signature(s) predicting their insertion in cell membranes. Some proteins in the surfome also have signal peptides that control topology but do not lead to secretion.

**Table 1:** MHC-II binding affinities of all overlapping 15-mers in the surfome of Staphylococcus aureus COL NC_002951.

MHC-II Supertype	Ave ln(ic50)	Std Dev ln(ic50)	10%-tile ln(ic50)	Ave IC50 (nM)	Ave-SD ic50 (nM)	10%-tile ic50 (nM)	Ave-2SD ic50 (nM)
**DRB1_0101**	4.48	3.11	0.54	88.27	3.95	1.72	0.18
**DRB1_0301**	6.29	1.93	3.81	540.59	78.15	45.28	11.30
**DRB1_0401**	5.31	2.59	1.95	202.23	15.12	7.04	1.13
**DRB1_0404**	5.23	2.76	1.63	187.57	11.84	5.12	0.75
**DRB1_0405**	4.38	1.90	1.92	79.92	11.96	6.81	1.79
**DRB1_0701**	4.29	2.84	0.62	73.33	4.27	1.85	0.25
**DRB1_0802**	7.05	2.00	4.48	1151.07	155.45	88.42	20.99
**DRB1_0901**	5.85	2.48	2.64	346.90	29.03	13.99	2.43
**DRB1_1101**	5.58	2.52	2.35	265.50	21.39	10.46	1.72
**DRB1_1302**	7.14	1.95	4.62	1257.67	178.85	101.68	25.43
**DRB1_1501**	5.86	2.74	2.31	351.12	22.61	10.07	1.46
**DRB3_0101**	8.26	1.95	5.74	3861.57	547.81	312.37	77.71
**DRB4_0101**	5.69	2.20	2.81	294.70	32.68	16.67	3.62
**DRB5_0101**	4.92	2.60	1.58	136.76	10.12	4.85	0.75

**Average**	**5.74**	**2.40**	**2.64**	**631.2**	**80.2**	**44.7**	**10.7**

**Exp(Average) nM**	310.5	11.0	14.1				

The surface proteome consists of all proteins that have one or more predicted transmembrane helices in their structure. The statistics were derived from approximately 216,000 15-mers for 14 supertypes, or about 3.02 million binding predictions. The NN were trained and the predictions were made in the natural logarithmic domain (ln). The statistical parameters are for the entire proteome, as this would constitute the population of peptides presented binding to MHC molecules on the surface of antigen presenting cells.

Prediction of B-cell epitopes, MHC-II binding, and topology for 15 strains of *Staph. aureus *(listed in Additional File 3, Table S3b) have been done. Predicted B-cell epitopes were found to be located inside and outside the bacterial cell membrane, but virtually none in the transmembrane domains, perhaps due to alpha helical structure of the transmembrane peptides. In the very few instances where extension into membranes did occur (<2%), the predicted B-cell epitope only penetrated a few amino acids. This may represent an error in the prediction of the edge of the transmembrane domain.

A summary of the topology of the proteins and the predicted MHC-I and MHC-II binding affinity of peptides in *Staph. aureus *COL is shown in Table 2. It is recognized that the immunological response to *Staph. aureus *should be mediated primarily through MHC-II; MHC-I is included for completeness. The table shows the relatively higher binding affinity of peptides found in membrane spanning segments of the proteome. The difference in the ln(ic50) of the MHC-II affinities of the intracellular and extracellular segments is small but is statistically significant.

**Table 2:** Characteristics of the surface and secreted proteome of *Staphylococcus aureus *COL

Total Proteins	with TMH	with TMH and SP	Secreted
2,615	649	69	186

**MHC-I**	**9-mers**	**Avg ln(ic50) of group**	**Total**

inside	48,222	9.3	238,320
membrane	51,643	8.4	
outside	138,455	9.3	

**MHC-II**	**15-mers**	**Avg ln(ic50) of group**	**Total**

inside	40,422	6.4	210,466
membrane	38,698	4.6	
outside	131,346	6.6	

Total proteins and protein topology and MHC binding characteristics to different protein domains of *Staph aureus *COL surface and secreted proteins. Peptides within transmembrane domains to have a significantly higher binding affinity to both MHC-I and MHC-II. The ln(ic50) means shown are for peptides in the dataset for which both their N-terminus and C-terminus in the predicted transmembrane domain. All means were different from one another by ANOVA p < .0001. TMH = transmembrane helix; SP = signal peptide.

### Interface with a Diverse Host Population

The array of genetic variants (supertypes) of HLA molecules in the human population vastly exceeds that for which there are peptide training sets. Additionally, and yet further increasing the combinatorial possibilities, is the fact that each individual has both parental genotypes of MHC on their cell membranes. Despite the combinatorial complexity, examination of the statistics of the predicted binding affinities to a number of different proteins in the proteome of *Staph. aureus *gave rise to several observations which suggested that it would be possible to derive a system for determining the probability of binding not only for single supertypes, but for a population of combinatorial supertypes for which a trained NN was available. The processes outlined above make it possible to put entire proteomes (or multiple proteomes) consisting of millions of binding affinities into a single data table, in a familiar spreadsheet interface on a standard workstation computer.

Table 3 shows the predicted binding affinities for each of the DRB supertypes in combination with each of the other DRB molecules (105 permutations) simulating heterozygous individuals (detail in Additional File 6). Inside an antigen presenting cell where peptides from a digested organism (e.g. *Staph. aureus *COL) are coming into contact with MHC-II molecules, those molecules with higher affinity (smaller of the two ln affinity numbers) would be expected to dominate in the binding process. One of the striking features that emerges from this table (bottom rows Table 3) is the overall advantage of heterozygosity. Individuals randomly inheriting combinational pairs of the 14 supertypes stand to have a higher binding affinity than if they had only one type. A second observation is the segregation of different alleles within the sorted list. The heterozygosity advantage and the 10 percentile threshold, being in a range considered a useful biological range of affinity, suggested the possibility of averaging over all genotypes as a means of predicting binding in a population of individuals carrying MHC-II molecules of unknown genotype on their cells (as would be the case in a randomly selected vaccinee population). These results suggest that combinatorial pairs of supertypes need to be considered in statistical selection and screening processes, for example in clinical trials.

**Table 3:** MHC-II binding affinity of heterozygous and homozygous pairs

	S1	S2	10%tile S1	10%tile S2	10%tile Average	10%tile min of pair
Top Ten	DRB1_0101	DRB1_0101	0.54	0.54	0.54	0.54
	
	DRB1_0301	DRB1_0101	3.81	0.54	2.175	0.54
	
	DRB1_0401	DRB1_0101	1.95	0.54	1.245	0.54
	
	DRB1_0404	DRB1_0101	1.63	0.54	1.085	0.54
	
	DRB1_0405	DRB1_0101	1.92	0.54	1.23	0.54
	
	DRB1_0701	DRB1_0101	0.62	0.54	0.58	0.54
	
	DRB1_0802	DRB1_0101	4.48	0.54	2.51	0.54
	
	DRB1_0901	DRB1_0101	2.64	0.54	1.59	0.54
	
	DRB1_1101	DRB1_0101	2.35	0.54	1.445	0.54
	
	DRB1_1302	DRB1_0101	4.62	0.54	2.58	0.54

Bottom Ten	DRB1_0301	DRB1_0301	3.81	3.81	3.81	3.81
	
	DRB1_0802	DRB1_0301	4.48	3.81	4.145	3.81
	
	DRB1_1302	DRB1_0301	4.62	3.81	4.215	3.81
	
	DRB3_0101	DRB1_0301	5.74	3.81	4.775	3.81
	
	DRB1_0802	DRB1_0802	4.48	4.48	4.48	4.48
	
	DRB1_1302	DRB1_0802	4.62	4.48	4.55	4.48
	
	DRB3_0101	DRB1_0802	5.74	4.48	5.11	4.48
	
	DRB1_1302	DRB1_1302	4.62	4.62	4.62	4.62
	
	DRB3_0101	DRB1_1302	5.74	4.62	5.18	4.62
	
	DRB3_0101	DRB3_0101	5.74	5.74	5.74	5.74

		**Mean**	**2.92**	**2.37**	**2.64**	**1.88**

		**Std Dev**	**1.47**	**1.41**	**1.07**	**1.08**

Ten percentile MHC-II binding affinity statistics for 105 different heterozygous and homozygous supertype combinations for 15-mer peptides from the surface proteome of Staphylococcus aureus COL. The results were obtained using 14 MHC-II supertypes for which training sets were available to train the NN. The surface proteome is defined as proteins that are predicted to have one or more transmembrane helices and are therefore expected to be inserted into the cell membrane. Top and bottom ten pairs are shown in this summary table; complete data set in Additional File 6.

**Figure 2:**
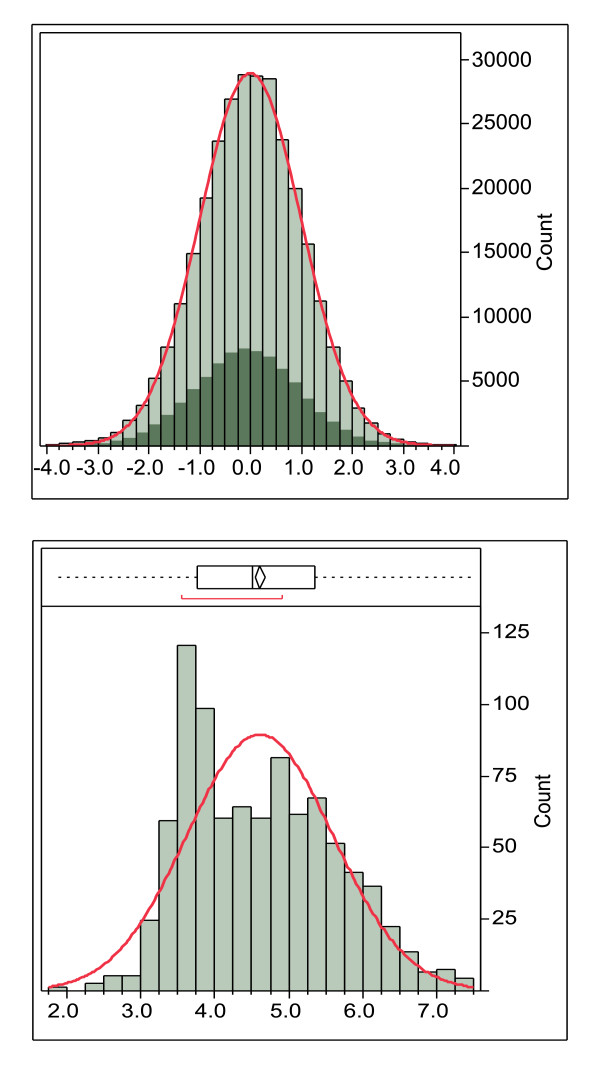
**Example of the global standardization process**. The global standardization process using DRB*0101 15-mers of the combined secretome and surfome from NC_002951 *Staphylococcus aureus *COL (289,760 peptides). The highlighted area shows the peptides with an N-terminal amino acid predicted to be in a membrane that is shifted from the mean in the original data but is coincident with the mean after standardization. Lower panel. Average lnN(ic50) of DRB*0101 15-mers done on a protein basis. In this case the histogram bars are the number of proteins with the indicated ln(ic50). The non-normal distribution is caused by proteins with transmembrane domains with higher binding affinity.

The different alleles and allelic combinations shown in Table 3 have significantly different means and variance and this complicates statistical analysis and thresholding. All of the proteins in the *Staph. aureus *surfome, comprising about 210,000 15-mers, were used in a global standardization process (summarized in Additional File 5). By using all the 15-mers in the proteome for standardization, the statistical processes are brought into line with the biological process where an engulfed foreign organism would be digested and the peptides presented would be the entire repertoire of the organism. Furthermore, the construction of normally distributed populations provides a means of rigorous and meaningful statistical screening and selection processes from normal Gaussian distributions (Figure 2).

A second distribution anomaly is shown in Figure 2. Not only does the binding affinity vary across different MHC alleles as described above, it also varies between proteins in a proteome, giving rise to distributions like that seen in Figure 2. This clearly demonstrates why any organism-level conclusions about binding affinity cannot be made based on measurements made on peptides in isolation and should preferably not be made on individual proteins. The global standardization process based on standardized ln(ic50) ranks all peptides and produces a ranking of affinities of all peptides in a proteome against all other peptides in that proteome. This is the situation that would arise as an infectious organism is digested in an antigen presenting cell.

### Correlations between MHC-I and MHC-II

**Figure 3:**
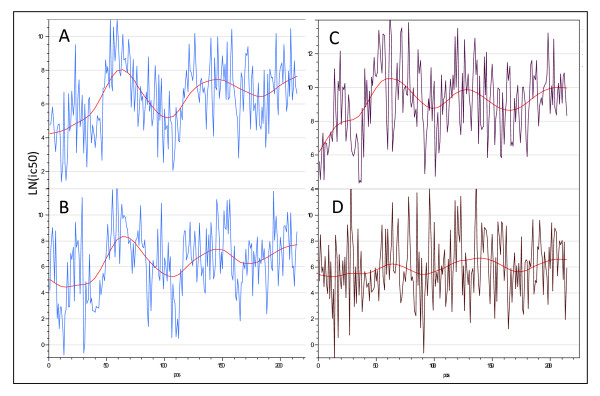
**Example of long range variation in mean MHC II affinity across a single protein (Thermonuclease precursor Staphylococcus aureus COL gi 57650135)**. (A) DRB4*0101 and (B) DRB1*0404 have a high correlation (r = 0.6) while (C) DRB3*0101 and (D) DRB1*0901 have a low correlation.

By examining the plots of many different proteins with different types of data portrayal we observed that, despite individual 15-mer peptides showing widely different predicted binding affinities for the different MHC supertypes, there was a tendency for high binding for all supertypes to locate in certain regions of molecules and low binding in other regions. This can be seen by undulations in the averaged mean affinities across a protein sequence. Not only was this the case among MHC-II supertypes, but was also seen with the overall means of all MHC-I and MHC-II supertypes (Figure 3; Table 4). After examining many different proteins individually it emerged that each protein has a characteristic undulation pattern regardless of the supertype or MHC. Computed on an affinity basis the variations are very large with the mean affinity varying over a thousand-fold for peptides from different regions within a protein molecule. This long-range variation is superimposed on a large peptide to peptide variation within the protein.

**Table 4:** Pearson correlation coefficient of ln(ic50) for pairs of MHC-I and MHC-II alleles.

	MHC-I			MHC-II		
Top Ten	**HLA 1**	**HLA 2**	**r**	**HLA 1**	**HLA 2**	**r**
	
	A*2402	A*2301	0.67	DRB4*0101	DRB1*0404	0.63
	
	B*4403	B*4402	0.60	DRB1*0701	DRB1*0404	0.63
	
	A*2403	A*2301	0.54	DRB1*1501	DRB1*0404	0.62
	
	B*4403	B*4002	0.51	DRB1*0405	DRB1*0404	0.58
	
	B*4501	B*4403	0.50	DRB1*1101	DRB1*0404	0.55
	
	A*2403	A*2402	0.49	DRB1*1501	DRB1*0701	0.54
	
	B*4403	B*1801	0.47	DRB5*0101	DRB1*1101	0.53
	
	B*4501	B*4002	0.45	DRB4*0101	DRB1*1501	0.52
	
	B*5301	B*5101	0.41	DRB5*0101	DRB1*0404	0.52
	
	B*5301	A*2402	0.40	DRB1*1101	DRB1*0802	0.51

Bottom Ten	B*5701	B*1801	-0.26	DRB3*0101	DRB1*0405	0.23
	
	B*4501	A*2601	-0.26	DRB1*1302	DRB1*0405	0.23
	
	B*5701	B*5401	-0.27	DRB1*1302	DRB1*1101	0.23
	
	A*3002	A*2301	-0.28	DRB1*0901	DRB1*0301	0.22
	
	B*4501	A*6801	-0.29	DRB3*0101	DRB1*0101	0.22
	
	B*4403	A*3002	-0.29	DRB1*0802	DRB1*0301	0.21
	
	B*4002	A*6801	-0.31	DRB3*0101	DRB1*1101	0.20
	
	B*4402	A*2902	-0.31	DRB5*0101	DRB3*0101	0.20
	
	B*4501	A*2301	-0.32	DRB1*0301	DRB1*0101	0.20
	
	B*4403	A*2902	-0.34	DRB3*0101	DRB1*0802	0.19
	
	B*4002	A*3002	-0.46	DRB3*0101	DRB1*0901	0.15

The Pearson correlation coefficients were computed for all allele combinations on a random sample of 1000 15-mers (MHC-II) and 1000 9-mers (MHC-I) from the *Staphylococcus aureus *COL dataset. All coefficients are significant p < 0.0001. Note that for MHC-I there are both negative and positively correlated pairs. Some of the coefficients ~0.0 were not statistically significant in this case.

It also became apparent that, despite the large differences in affinities between peptides, for a particular peptide some of the ic50 values were highly correlated across MHC alleles. The Pearson correlation coefficients for the top ten and bottom ten pairwise comparisons are shown in Table 4. For MHC-II all of the pairwise correlations are statistically significant and positive, though of varying magnitude. For MHC-I there is a subset that is positively correlated, another that is negatively correlated and a third group of non-correlated alleles.

### Windowing of High Affinity Binding

The positive correlations among MHC alleles and the other statistical characteristics led us to experiment with methods of computing binding metrics that encompassed a population of heterozygotic combinations; effectively a population phenotype.

**Figure 4:**
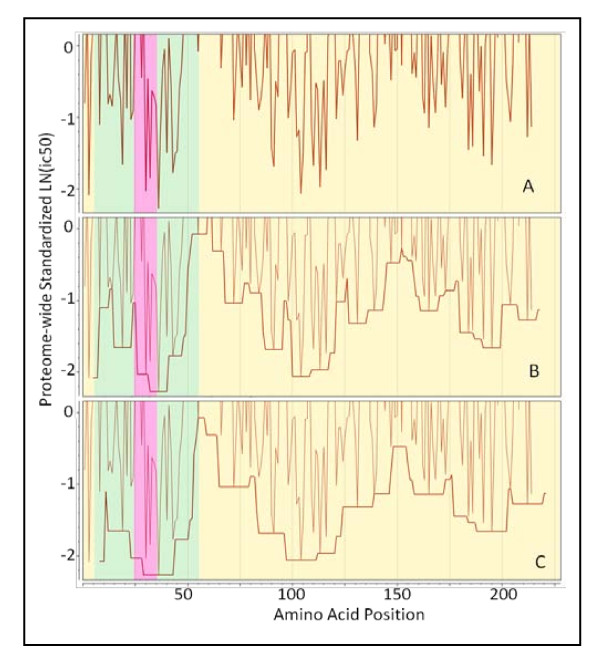
**Demonstration of application of a binding window around a high affinity binding 15-mer**. (A) Actual standardized binding affinity N-terminus of the 15-mer begins at the point plotted. (B) peptide movement window ± 4 amino acids and (C) a binding window of ± 7 amino acids. The fine line in panels B and C is identical to that plotted in A. Semi-transparent colors yellow = extracellular, green = transmembrane domains, and pink = intracellular predicted by Phobius. Protein: Thermonuclease precursor *Staphylococcus aureus *COL gi 57650135.

From an immunological perspective the low affinity peptides are irrelevant; it is more useful to calculate a running average of high affinities in a way that captures the undulations in binding affinity across a protein sequence, while also capturing the population phenotype. Based on these concepts we developed a system to compute an average of standardized affinities for the permuted pairs for all supertypes within an adjustable (filtering) window. The window is defined as a stretch of contiguous amino acids positions over which averaging was carried out. Various windows (filtering stringencies) were tested, but the most useful smoothing was achieved with a window of ± half the size of the binding pocket (± 4 amino acids) as shown in Figure 4.

For MHC-II this is reasonably simple to envisage, as the ends of the pocket are open and peptides longer than 15 amino acids could undergo rapid association:dissociation "jiggling" until the highest binding configuration is found. In practice, the range of affinity constants in a pool of peptides may be as much as 1000 fold so that higher affinity peptides will very rapidly occupy the MHC binding site and remain bound in place. For MHC-I, with closed ends on the binding pocket, the possibilities are more limited.

Another factor, which has not been included in the predictions at this point, is the effect of the differential proteolysis that will contribute to the variable lengths of peptide with a possibility to interact with a binding pocket. Several tests of the potential impact of proteasomal cleavage were carried out with the webserver NetChop 3.1 at CBS on sample protein sets (not shown). From those experiments it appears that peptides are very likely to be cut into pieces shorter than 9 amino acids, so that the MHC-I presentation of a peptide is the result of interception and capture by the MHC binding reaction during proteolytic cleavage. The patterns of proteolytic cleavage of proteins by lysozomal enzymes suggest that they would be equally aggressive and that peptide processing for MHC-II presentation would be expected to be comparable.

### Permuting Windows for the Population

The permuted minima within the window described above (and shown in Figure 4) are averaged to arrive at a single number for all MHC-I allelic combinations and another for all MHC-II combinations for each particular amino acid position. Through experimentation we found that this process produced metrics whose undulations tracked the visually obvious patterns of MHC binding in proteins (as seen in Figure 3). As the numbers were drawn from a standardized dataset the resulting sample were also normally distributed albeit at a distance (negative) from the population mean as a whole. Thus, statistical thresholds could be applied to these metrics that were based on normal populations. Each allelic combination was given an equal weight. This is a generalizable concept; it is also possible to compute the predicted population phenotype for various subpopulations using appropriate weighting for genetic frequencies. The windowing operation is not dependent on standardized populations and the actual ln(ic50) can be used as well to compute a running average of binding affinity for any single allele.

The output of these computational processes was tabulated in a master database for the organism (Figure 1, Step 13). Selected coincident epitope groups comprising regions of proteins where peptides met three criteria were determined. In these both binding threshold for MHC and the B-cell epitope probability threshold were in the 10 percentile range and the run of amino acids in the predicted BEPI peptide was ≥4 amino acids. Selection of the 10th percentile in two characteristics in normally distributed variables on a probability basis should be a product of two probabilities or about a 1% coincidence where MHC binding regions overlapped either partially or completely with predicted B-epitope regions.

### Graphical Display

**Figure 5:**
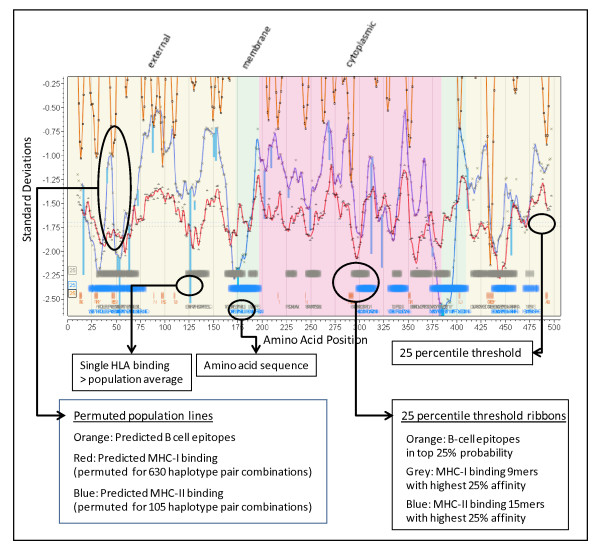
**Annotated multidimensional overlay graphics of integrated analysis**. *Cryptosporidium parvum *(Iowa II) hypothetical protein cdg5_540. GI: 126649159 as an example. The portion of the overlay graphic shown contains annotations related to various cellular features and protein topology well as the standardized predictions critical to the immunological recognition of the protein. This provides a graphical means of visualizing a multidimensional database of related information. At various magnification levels actual peptide sequences can be visualized as well as experimentally mapped locations for any desired HLA molecule.

Figure 5 shows an annotated example of the graphical output from the system we have described above.

### Examples of Retrospective Comparisons

#### AntiJen multi species benchmark dataset

A summary table of our findings on the AntiJen dataset and representative examples of graphical output for a cross section of these proteins is provided in Additional File 2, Table S2b and Additional File 2, Figure S2c.

The AntiJen data set comprises proteins from viruses, bacteria, protozoa, mammals, plants, and a number of other sources. Some are surface proteins. B-cell epitopes were found to be located predominantly in the external surface loops, and to a lesser degree in the cytoplasm. As with *Staph. aureus*, we observed CEGs in all types of proteins. A large percentage (>20%) of B-cell epitopes were affiliated (i.e. overlapping or with their borders within 3 amino acids) with one or more MHC-I or MHC-II high affinity binding peptides. Over 78% of MHC-I high affinity binding peptides were affiliated with one or more B-cell epitopes, as were >95% of MHC-II binding domains. MHC-I and MHC-II high affinity binding domains tended to be affiliated with each other.

Predicted epitopes were more prevalent in membrane associated proteins. Many proteins, particularly those with no transmembrane regions, had quite sparse epitope distribution, nevertheless CEGs were observed in most.

#### Staphylococcus aureus

We have completed analyses of B-cell epitopes, MHC-II binding, and topology for 15 strains of *Staph. aureus *(strains included are listed in Additional File 3, Table S3b). Table 5 summarizes the results of the analysis, based on predicted average minimum binding permuted for an immunogenetically diverse heterozygous host population. The overlaps of the various epitopes were computed using a SOM algorithm on the centroids and dispersions of the predicted epitope segments.

**Table 5:** Summary of analysis of *Staphylococcus aureus *strains

Output for *Staph aureus *COL	Metric
No. of Proteins in *Staph aureus *Col proteome	2615

No. of Surface Proteins (with Transmembrane Helices)	649

Number of Proteins with Signal Peptides	255

Sub-proteome analyzed (secreted and membrane affiliated)	835

Total B-cell epitopes > 4 aa long	14,089

Total B-cell epitopes overlapping or borders within 3 aa of a MHC-II high affinity binding peptide	4,527

Percentage of B cell epitopes overlapping or bordering within 3 aa of a MHC-II high affinity binding peptide	32.13%

Total MHC-II high affinity binding peptides	3,230

**Output for 15 Strains of *Staph. aureus***	**Metric**

Proteomes of *Staphylococcus aureus *strains analyzed	15

Unique CEGs detected (all strains)	5646

CEGs conserved in 15/15 strains	572

Additional CEGs conserved in 14/15 strains	364

Median CEG (amino acids)	25

Minimum CEG (amino acids)	15

Maximum CEG (amino acids)	60

Proteins conserved in 15 strains with conserved CEGs (secreted and membrane affiliated)	140

*Staphylococcus aureus *COL is used as an example of the properties analyzed and observed within a strain. The data from 15 strains of *Staph. aureus *is summarized in the lower half of the Table. The strains are listed in Additional File 3, Table S3b. Epitopes conserved in 14 of 15 strains tend to have only a single amino acid change. Epitopes characterized as B cell epitopes are within the top 25% on a permuted population basis. Peptides characterized as a MHC-II high affinity binding peptide are n the top 25% of binding affinities on a permuted population basis as defined in Table 4. A CEG is a coincident epitope group comprising a stretch of amino acids where overlapping or adjacent B-cell epitopes as well as MHC high affinity binding peptides are predicted.

Of all predicted B-cell epitopes in *Staph. aureus *COL strain, 32.13% were found to be overlapping or affiliated with MHC-II high affinity binding peptides; 66.54% of MHC-II binding peptides were found in affiliation with B-cell epitopes.

Within the 15 strains of *Staph. aureus *we mapped a total of 5646 CEGs. Of these, 572 were conserved across all 15 strains. A further 364 were found in 14 of the 15 strains with usually only a single amino acid change in the one non-conserved strain. Of the approximately 2615 proteins making up the proteome of each of the 15 strains, 98 proteins within the surfome and a further 42 proteins in the secretome were conserved across all strains and thus contained conserved CEGs.

**Figure 6:**
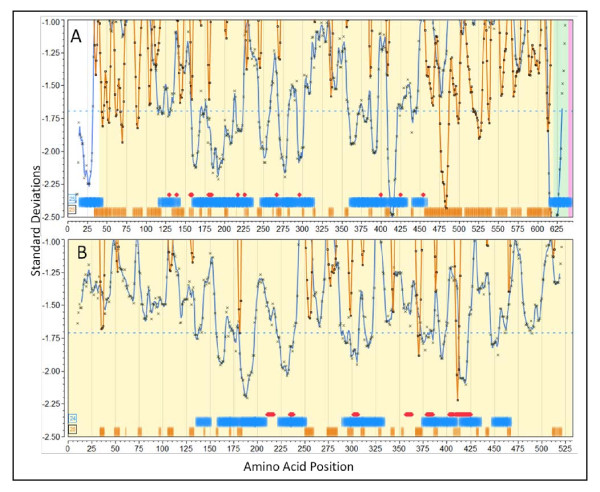
**Predicted and experimentally mapped epitope regions for two proteins from *Staphylococcus aureus *COL (NC_002951)**. Graphic overlay using proteome-wide standardization of scoring metrics. Blue line is the predicted population phenotypic MHC-II binding. This is computed as described in the methods for fourteen MHC-II alleles permuted as dizygotic combinations within a window ± 4 of the amino acid position indicated. The orange line is the predicted B-cell epitope probability for the particular amino acid being within a B-cell epitope. Actual computed data points are plotted along with the line that is the result of smoothing with a polynomial filter [49]. Blue horizontal bands are the regions of high probability MHC II binding phenotype and orange horizontal bars are high probability predicted B-cell epitope regions. The percentile probabilities used as the threshold are as described in the text and is indicated in the number within the box at the left. The red diamonds (or groups thereof) are experimentally mapped regions of Ig binding. The experimental mapping is described in more detail in Additional File 3 Table S3a. (A) LPXTG cell wall surface anchor protein, IsdB (GI:57651738) [51]. (B) ABC transporter, ATP-binding protein (GI:57651892) [52].

To evaluate our observations alongside the experimental findings of others, we identified a number of publications which provide characterization of epitopes within five *Staph. aureus *proteins, documenting both predicted B-cell epitopes and MHC binding. In Additional File 3, Table S3a we tabulate the correlation between our observations of CEGs and the experimental findings for these. Much of the data pre-dates genome sequencing projects and thus reconciling the literature with Genbank is challenging. For example, in some cases peptides were reported with amino acid numbering without consideration of the signal peptide cleavage in the Genbank proteome repository. In some cases, such as with the staphylococcal toxins, the cleavage is substantially distal from the starting methionine. Figure 6 shows the graphical plots with the published experimental results overlaid for two proteins; additional plots are found in Additional File 3, Figure 3c and Figure 3d. We caution that our plots show the permuted human population average positions for MHC-II binding; publications may report on experimental results derived from single HLA or mouse MHCs. Overall there is remarkable correlation. Where the most detailed fine epitope mapping is compared to our prediction the mapped contact points either overlap or are within 5 aa. In other cases where mapping is not so detailed the overlapping is extensive. We have predicted additional high affinity MHC-I and MHC-II binding peptides not identified in the literature.

#### Staph. aureus Iron Regulated Determinant B (IsdB) NC_002951.57651738

The *Staph. aureus *protein IsdB is a vaccine candidate; recent papers characterize its immunological features [50,51]. In Figure 6A predicted regions of MHC-II binding peptides and predicted B-cell epitope regions are shown along with the positions at which point mutations abrogated monoclonal antibody binding [51]. Several features are noteworthy in the patterns seen, as they appear commonly in proteins we have reviewed. First, the population permuted high affinity binding regions vary by over 2 standard deviation units. This corresponds to over a 1000-fold range in average highest predicted affinity binding. Regions where predicted high affinity human MHC binding peptides are absent sometimes extend over several hundred amino acids (see also *Staph. aureus *Protein A, Additional File 3, Figure S3d). Second, regions with a paucity of high affinity MHC binding peptides tend to have long stretches of high probability B-cell epitopes. All the regions of monoclonal antibody contacts mapped by Brown *et al *[51] are located in regions of where the population would be predicted to have high affinity MHC-II binding and the alignment with the predicted B-cell epitopes is strong. It should be pointed out that the 25th percentile (standardized) B-cell probability threshold is twice the stringency recommended by the BepiPred server; using a lower stringency resulted in all the mapped B-cell epitopes overlapping the predictions. The muteins produce a fine map of discontinuous epitopes and, thus, while the B-cell epitope prediction algorithms can only predict individual linear peptide probabilities, it appears that they reliably predict sub-segments of discontinuous epitopes.

#### Staph. aureus ABC transporter protein NC_002951.57651892

Figure 6B shows the predicted and mapped patterns for an ABC transporter protein. This protein was identified as a target for clinical immunotherapeutic development based on the work of Burnie *et al *[52], which documents antibodies recognizing this protein in the sera of patients with staphylococcal septicemia. Unfortunately, neither the HLA of the patients, nor the strains of *Staph. aureus *were documented. There are several families of this protein and they are present in all strains of *Staph. aureus*. It is predicted by virtue of its signature motif as an ATP transporter to be a membrane protein. We include proteins of this sort in our surfome dataset by the use of PSORTb [53] regular expression motif identifiers. We found the equivalent protein in all *Staph. aureus *currently available to be in two groups differing by two point mutations. A notable feature of the ABC transporter is that all the mapped monoclonal antibody binding regions are in areas predicted to have high affinity MHC -II binding. Two caveats about the results point to a common issue when attempting to reconcile predictions with older studies. The mapped regions were selected for more detailed study by Burnie *et al *[52] simply because the ELISA results for the peptides were more than two standard deviations above the mean. Hence there is not a complete panel of experimental results to compare the B-cell epitope predictions. Clearly the experiments would also have produced a number of peptides over 1 standard deviation etc. but those were not reported. As a result, the curators of the IEDB have classified all other peptides in this protein as "B-cell epitope (true negative)". No MHC binding results were reported.

#### Vaccinia

**Figure 7:**
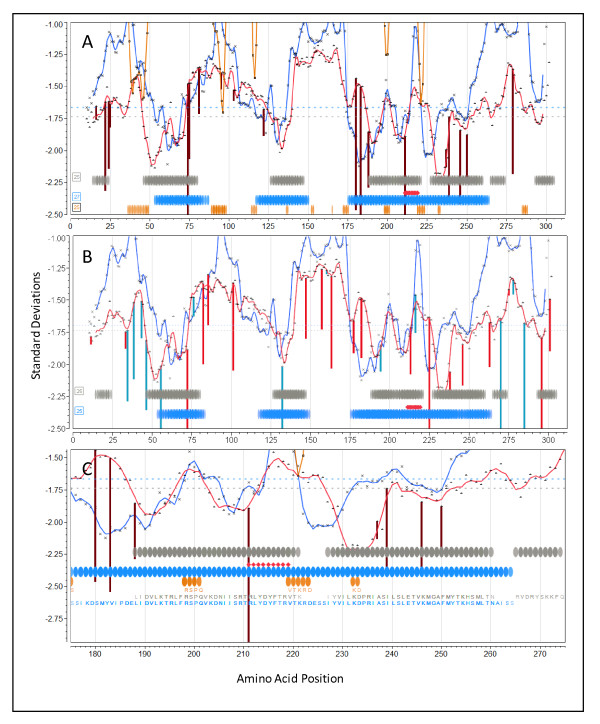
**Overlay epitope maps of locus I1L (GI:68275867) from Vaccinia virus Western Reserve**. (A) Vertical lines (dark red) are the N-terminal positions of predicted high affinity binding 9-mer peptides for A*0201 predicted by neural net regression. (B) Vertical lines are the N-terminal positions of predicted high affinity binding 9-mer peptides for A*1101 (red) and B*0702 (blue) predicted by neural net regression. (C) Higher resolution showing fine detail of A*0201 mapping. In all three panels the experimental overlay is for MHC I 9-mer peptides mapped in HLA A*0201/K^b ^transgenic mice [55]. Symbols as described in legend to Figure 6. Background is unshaded because this protein is predicted to lack any membrane domains.

The complete proteome for VACV Western Reserve was downloaded from Genbank and processed as described above. We generated graphical output for all the proteins and then compared the output for proteins reported as containing immunodominant binding T-cell epitopes [54,55]. Figure 7 shows graphical output for I1L (GI:68275867). Additional File 7 shows comparable output for proteins A10L (GI:68275926), A14L (GI:68275930), and A17L (GI:68275934).

The experimental studies by Pasquetto [55], to which we made comparisons, were done in transgenic mice carrying human MHC-I molecules. Thus they represent perhaps the most clear attempt to match *in silico *predicted to experimental human MHC binding. Figure 7 depicts plots for protein I1L shown at two different magnifications, to enable the visualization of peptide sequences in the overlays. As I1L lacks transmembrane domains the background has been left uncolored. The colored vertical lines indicate the specific location of the leading edge (N-terminus of a 9-mer) of predicted high affinity peptides for the particular indicated HLA. The colored lines extend below the permuted population average and indicate that specific HLA shows higher affinity binding for that peptide than does the population as a whole. Also shown are the locations of predicted B-cell epitopes. Notably, the peptides experimentally mapped by Pasquetto *et al *[55] (and shown in Figure 7 by red diamonds) are ones with predicted binding affinity of at least 2.5 standard deviations below the mean.

Protein I1L was reported to also contain a B-cell epitope and led to the suggestion that B-cell and T-cell epitopes being deterministically linked within the same protein [7]. Based on the permuted population phenotype, we predict MHC-I and MHC-II high affinity binding peptides, and multiple B-cell epitopes, affiliated in three CEGs. The predictions for each HLA used in transgenic mice by Pasquetto [55] were examined. HLA-A*0201 (Figure 7A and at higher resolution in 7C) shows a peak of very high affinity binding for the aa 211-219 peptide RLYDYFTRV, a remarkable 3.95 deviations below the mean. The predicted initial amino acid of this peak binding coincides exactly with the initial arginine in the 9-mer described by Pasquetto [55]. Interestingly, we also predict that HLA-A*0201 mice should detect binding of a similar high affinity starting at amino acid 74. As there are ten B-cell binding regions in the top 25% probability, any one or a combination of these could account for the linked epitope response noted by Sette *et al *[7], however a group of three predicted B-cell epitopes lie within positions 198-233. Figure 7B shows the binding affinities predicted for HLA-A*1101 and HLA-B*0702. There are also high peaks of affinity, but not coincident with those of HLA-A*0201.

## Discussion

### Epitope Analysis System

We describe an integrated epitope analysis system which is based on multi-dimensional and orthogonal physicochemical properties of sequences of amino acids using a multilayer perceptron neural net to conduct QSAR regression predictions for peptide affinities to 35 MHC-I and 14 MHC-II alleles. The system allows rapid processing of single proteins, entire proteomes or subsets, as well as multiple strains of the same organism. It allows consideration of diversity of both microorganisms and of host immunogenetics.

The program can be used to predict B-cell epitope peptides and MHC-I and MHC-II binding peptides, across strains or unique to one strain of organism, plus spatial and topological correlation of membrane proximity. It predicts peptide affinities for HLA supertypes, heterozygous pairs and population-permuted heterozygous pairs.

The system is built on JMP^® ^(and JMP^® ^Genomics) data visualization and statistical platform framework and configured to run on a desktop computer and generate graphical and tabular outputs. The predictions can be expanded to other MHC molecules as more MHC training sets become available.

We have tested the system retrospectively against proteins from two organisms for which epitopes have been documented independently by many labs and have used the 'AntiJen' benchmark data set. The approach we describe has performed well for a wide variety of prokaryotic and eukaryotic proteins, including mammalian cellular surface and secreted proteins. The graphical visualization output allows perception of patterns among predicted epitopes heretofore not recognized. Just as GIS have permitted a more integrated view of landscapes and have provided insights which have aided land use and public policy decisions, the layering and integration of all available immunological and topological information offers new insights into organization in the immune response.

### Epitope Patterns

Having applied the integrated analysis system to the test data sets, as well as to other proteomes, a number of patterns emerged. Furthermore, the ability to visualize coincident features within proteins brings a new level of insight into possible functional interactions.

#### Concordance in Binding Affinity on a Peptide Basis Across HLAs

The initial core observation was that there is long range coincidence of predicted high affinity MHC binding peptides among different HLA's in a population. When a given protein is broken down into sequential peptides (9-mers or 15-mers) and binding is compared against an array of all HLAs, it is possible to map areas of higher and lower predicted binding affinity and there is general concordance between HLAs. Distribution is non-random and areas of high binding can be defined on a population basis. We observed closer positive correlation within MHC-IIs at any given peptide than within MHC-I. This non-random distribution and areas of high binding affinity make it possible to plot a population phenotype binding affinity (using the permuted pairs) and accurately predict peptides which will serve as high affinity MHC binders for the population at large, given that all have the benefit of heterozygous alleles (e.g. in mass vaccination). We have designed the system to permit variation of the stringency in evaluating binding affinity, but have focused on the top 25% binding affinity.

While there is remarkable general coincidence between HLA alleles for the optimal binding positions when viewed as a population, there are subtle - and sometimes not so subtle - differences in highest affinity peptide binding position of alleles when examined singly. These differences between individual HLAs point to associations of HLA with a number of diseases, and in particular the opportunity to construct personalized vaccines with knowledge of the patient's HLAs. It also has implications when isolated peptides are selected as subunit vaccines for a diverse population and for the design of clinical trials representative of HLA alleles. Reassuringly, heterozygosity offers a distinct advantage in T-cell epitope presentation, bringing new meaning to the survival of the "fittest".

#### Regional Correlations Within Proteins

When the graphical plots for complete proteins are viewed, other patterns emerge. Many predicted MHC-II and MHC-I binding patterns show minima in the same region. Both MHC classes appear to sample the same "epitope structural space" on the protein, although not necessarily at the same time or in the same location in an antigen presenting cell.

Epitope clustering observed by others [56] appears to take on a more systematic organization when viewed as an integrated map. Our predictive analysis of the multi-species benchmark dataset, and the other organisms we have examined, lead to the conclusion that there are three groups of immunogenic peptides/polypeptides:

a. Peptides which comprise B-cell epitopes overlapping with or in close proximity (within a few amino acids) to peptides binding with high affinity to MHC-I or MHC-II molecules, which we have called CEGs;

b. Those containing B-cell epitopes only;

c. Peptides which bind to MHC-I or MHC-II molecules, and which are not associated with B-cell epitopes.

The first two groups of epitopes (a) and (b), comprising B-cell epitopes, are found external, and to a lesser degree internal to cell membranes. Virtually no predicted B-cell epitopes are mapped within membranes. Group (c) comprising MHC molecules without B-cell epitopes includes some of the highest affinity MHC binding regions located in membranes (Table 2).

The associations between predicted MHC binding peptides and predicted B-cell epitopes in CEGs are not a random event. In the *Staph. aureus *dataset approximately a third of B-cell epitopes have associated MHCs (>20% in AntiJen proteins), and over two-thirds of MHC high affinity binding regions have affiliated B-cell epitopes.

B-cell epitopes without close MHC binding regions could be components of more complex epitopes as the result of folding or positioning in the membrane. Thus what appear as isolated B-cell epitope sequences may actually be physically associated with components of B-cell epitopes in CEGs. Alternatively, they may act alone as T-independent antigens. Staphylococcal protein A is one example of a peptide which functions under some circumstances as a T-independent B-cell antigen [16]. Additional File 3, Figure S3d shows the remarkable B-cell epitope, but also the pattern of predicted MHC binding peptides in this protein.

We have shown that there is a very significantly higher predicted binding affinity among those MHC-II found within membranes relative to those outside or inside of cells. Some of the highest predicted MHC binding affinity peptides are those located within membranes. Epitopes mapped in proteins A17L and A14L in vaccinia are examples of such peptides.

#### Distribution of Peptide Binding Affinity within a Population

Binding of peptides to HLA molecules is a competitive process. The distribution of binding affinity determines which peptides are most likely to bind. Distributions of ln(ic50) binding affinities on a protein basis are not normally distributed across alleles (Figure 2). Some alleles approach a normal distribution but many show a bimodal distribution (i.e. peptides tend to be high binders or low binders, rather than medium binders). This characteristic makes standard statistical sampling challenging. Whether or not a protein has a transmembrane domain, some peptides are simply high affinity binders. Standardization makes it possible to establish thresholding criteria based on normally distributed populations and joint-probability distributions.

Using normalized distributions it is possible to compute population phenotypes which effectively "capture" the intra-protein regional correlations among MHC alleles. In practical terms, only high affinity matters, so we developed a scheme for computing a running average minimum over a variable amino acid "window" (Figure 3). In the population patterns using the whole proteins plots (e.g. Figure 6) we have used unweighted means, i.e. we have assumed all HLA alleles to be equally represented in the population. It would be possible to calculate phenotypes for various sub-populations by changing allele weighting. This might be appropriate, for instance, if selecting a vaccine for an ethnically isolated population.

#### B-cell Receptor Prediction Programs

B-cell predictive programs like BepiPred, and the principal component-based analogue of this we have developed, are likely to simply predict regions with physicochemical characteristics that lead to surface exposure and hence which form sites accessible for immunoglobulin attachment as predicted first by Hopp and Wood, and Parker [24,25]. The correlation with the fine mapping of the discontinuous epitopes for *Staph. aureus *IsdB [51] provides strong support for the concept that the B-cell epitope predictors identify linear sub-regions of discontinuous epitopes in proteins. We can speculate how, by hypervariable region mutations during antibody maturation, successive mutations might give rise to new molecules binding to additional short regions in the same vicinity and thereby lead to higher binding affinities for the new antibody mutein. By this concept a discontinuous epitope is a logical outcome of stepwise mutations during the maturation process. The reported "under performance" of B-cell epitope predictors [26] may be attributable at least in part to characteristics like those we map in *Staph. aureus *Protein A (region aa 325 to aa 475) and IsdB (aa 460 to aa 610). This characteristic pattern is seen frequently in proteome-scale overlay comparisons.

#### Comparative Standards

In evaluating a new predictive analytical system, the key question is how accurately does it predict? Which begs the question "relative to what gold standard?" Given the multidimensionality of the interface between host immunogenetics and pathogen, a "gold standard" has to be specific to the combination of HLA and peptide. Furthermore, measurements of binding affinity which has been derived using peptides in isolation from competition from the rest of the protein/proteome are of limited utility. In light of this we do not believe a standard four quadrant (true pos, false pos, true neg, false neg) scoring system is achievable at present. There are presently few examples in the literature (except perhaps Brown *et al *[51]) where the mapping of B-cell and T-cell epitopes has been done with sufficient detail to actually fill in the scoring quadrants necessary to compute an AROC. The three different types of epitope patterns mentioned above further complicate any attempts at developing a simple scoring system. Our prediction represents the theoretical maximum at any particular chosen statistical threshold. The higher the threshold stringency the fewer peptides that will meet the criteria.

In practice a number of factors limit the number of peptides which actually serve as epitopes. Timing of expression, or expression only under certain environmental conditions, is one filter [11]. A further major determinant is the rate of proteolysis, first to make the peptides available for binding, and secondly to degrade them into subunits below the threshold of recognition of MHCs. As with epitope binding, enzyme action on the proteins of the organism as a whole is a competitive process. Proteolytic cleavage is a critical process which determines which peptides are available to be bound by MHC molecules and hence displayed on cell surfaces as potential T-cell epitopes, and when such peptides become available or cease to be available due to further digestion. A peptide may have a predicted high binding affinity to an MHC protein, but if it contains a protease cleavage site precluding binding, it may never be presented at the cell surface. This is a factor not yet integrated into the analytical system we describe primarily because the cleavage training sets one would need to produce reliable neural net predictions for the relevant proteases are not available.

### Functional Implications of Patterns Observed

B-cell recognition gives rise to an antibody response. MHC-peptide binding is an intermediate step to presentation of MHC-peptide complexes for T cell recognition. We can speculate on whether a CEG represents a functional as well as a physical association. Given the consistency of the pattern, a functional association appears likely in which B-cell binding leads to uptake of the adjacent, overlapping or identical peptide to yield high affinity MHC binding peptides (via MHC-I or MHC-II), and, once bound, the peptide-MHC complex can lead to a productive T-cell response.

When viewed in the light of the observations of Batista of B-cell attachment and "pinching off" of surface segments during the formation of an immune synapse [15], one can envision how this physical proximity might facilitate the internalization of a peptide with a competitive advantage as a high affinity MHC binder. The dual presentation of B and T-cell epitopes would be consistent with the B-cell-T-cell interaction proposed by Lanzavecchia [6]. Similarly, preservation of B-cell epitopes by dendritic cells [57-59] would lead to the preservation and delivery to B-cells, not only of B-cell epitopes but also of overlapping MHC binding peptides.

The concept of specificity runs throughout the immunology literature. Generally specificity implies an absolute lock and key relationship. Rather, we see a pattern consistent with the dynamic competition for higher affinity MHC binding positions within the constraints of each set of unique host-pathogen interactions. Confronted with peptides from a different protein or different organism, the competition among peptides for binding to the same MHC molecules would simply be expanded. However, as affinity may range over binding constants of 1000 fold or more, there is a marked difference between high binders and weak binders. This is not inconsistent with a degeneracy in the binding specificity of peptides to MHC molecules. What matters is simply the ability of an MHC molecule to bind with high affinity a peptide representing the microorganism (or other immunogen) presented at that moment. The same MHC could just as well bind a peptide from another unrelated immunogen, not present in the same cell at that time. Given this multi step process, the effective "specificity" of the adaptive immune response is the product of multiple sequential binding affinities - {B-cell epitope binding }x {MHC binding} x{T-cell receptor binding to MHC-peptide complex}. No one of these steps needs to confer complete "specificity" but the combination increases the uniqueness or specificity of the antigen-immune response, essentially as a combination lock.

We do not speculate on the downstream implications of T-cell epitope binding, but note that T-cell stimulation may have both up and down-regulation effects and both positive and negative cytokine mediated feedback loops. Whether MHC binding regions located in CEGs have different roles from those MHC binding regions which are unrelated to B-cell epitopes is unknown. High affinity MHC binding regions located in membranes may be hidden until internalized and released by proteolysis, as proposed by Benacerraf [3]. This could occur through internalization of whole organisms or when such peptide fragments are "towed along" by internalization of adjacent tethered peptides.

It is of interest that we predict that a large percentage of predicted MHC-I high affinity binding peptides are coincident with those which bind MHC-II well. This implies that the precise intracellular pathway, and hence which proteolytic machinery the peptide encounters, may allow such peptides to stimulate T_CD8 _or T_CD4_, simultaneously or sequentially. Others have noted the need to understand the degree of overlap in the peptidome leading to stimulation of each pathway [60].

Given the apparent frequent overlap of B-cell epitopes with MHC binding regions, we suspect the literature contains observations on many peptide epitopes where, depending on what the experiment was designed to observe, function as either B-cell or T-cell epitopes has been described. Vaughan [61], in reviewing the literature on epitope characterization for *Plasmodium*, noted that 14% epitopes characterized by some workers as B-cell epitopes are reported by others to be T-cell epitopes, a percentage not dissimilar from that seen in the surfome of *Staph. aureus *and the AntiJen dataset.

At higher resolution, the fine structure of the diverse binding patterns of different HLAs shown (Figure 7) in vaccinia (and observed in other proteins; unpublished data) may shed light on the concept of immunodominance, in which immune responses by an individual are directed to a few peptides [60]. At one level it underscores the utility of HLA transgenic mice as indicators of immunodominance (binding affinity) for humans [54], while also calling into question extrapolation of peptide level MHC binding evaluations conducted in murine inbred strains and raising questions about the need to reflect immunogenetic diversity in clinical trials. Taking a broader perspective, if each HLA shows highest binding to a very narrow peptide sequence in given region of a protein (immunodominant peptide) it may represent a risk of microbial escape mutants [60]. However, because other HLAs bind to different adjacent peptides with high affinity, heterozygosity provides a backstop. Viewed in an immunogenetically diverse population, it provides a possible survival strategy in which any one escape mutant does not threaten an entire host population.

Overall, the predicted patterns which emerge from mapping imply yet greater coordination and organization of B-cell and T-cell responses that have heretofore been recorded. We do not underestimate the gap between prediction and experimental testing; however the ability to envision hypothetical functional interactions is a necessary precursor to design of experimentation.

## Conclusions

We present a predictive bioinformatics model which provides a means of rapidly analyzing whole proteins or proteomes and predicting a large number of B-cell peptide epitopes and high affinity MHC binding regions indicative of T-cell epitopes. The model also permits correlation of peptide epitopes with topological features. A visualization system of graphical overlays enables ready appreciation of the potential interplay of the features identified. The model has broad applicability to a wide array of proteins and demonstrated its performance with several well documented proteins.

Patterns seen in examination of a protein dataset derived from many organisms and with *Staph. aureus *and vaccinia show a consistent pattern of frequent coincidence of B-cell epitopes with MHC high affinity binding regions, suggesting that the physical proximity of B-cell epitopes to peptides with high affinity for MHC-I and MHC-II may be the norm, and of functional significance. This hypothesis remains to be further tested experimentally.

The data presented here for *Staph. aureus*, vaccinia, and the proteins in the AntiJen data set are of course a retrospective look at experimentally mapped epitopes with a view to validation of the integrated analysis system we have developed. We have processed a number of other proteomes and the challenge now begins as to how best to put the system to work as a prospective tool to support vaccine and antibody design and to provide better understanding of the immune response.

## List of Abbreviations

AROC: Area under the receiver operator characteristic curve; CBS: Center for Biological Sequence Analysis; CEG: Coincident Epitope Group; GIS: Geographic Information Systems; ic50: Inhibitory concentration 50%; IEDB: Immune Epitope Database; NIPS: Near identical protein sequence; QSAR: Quantitative Structure Activity Relationships; SOM: Self-organizing map.

## Competing interests

Drs. Bremel and Homan are founding scientists and employees of ioGenetics LLC. Patent applications have been filed on components of the technology described herein.

## Authors' contributions

RDB designed and performed computational analysis. Both authors conceived the study design, contributed to its execution, and drafted the manuscript. Both authors read and approved the final manuscript.

## Supplementary Material

Additional File 1 **Listing of internet sites with relevant computing and resource sites (PDF)**. Click here for file

Additional File 2 **AntiJen data (PDF)**. S2a. Table of proteins in AntiJen set. S2b. Summary table of analytical results. S2c. Representative graphics from AntiJen set. Click here for file

Additional File 3 ***Staph. aureus *data (PDF)**. S3a. Table of epitopes mapped experimentally in *Staph. aureus*. S3b. Strains of *Staph aureus *analyzed. S3c. Maps of three *Staph. aureus *toxins. S3d. Map of *Staph. aureus *Protein A. Click here for file

Additional File 4 **Process description (PDF)**. Click here for file

Additional File 5 **Global standardization criteria (PDF)**. Click here for file

Additional File 6 **Complete dataset summarized in Table 3 (PDF)**. Click here for file

Additional File 7 **Vaccinia additional figures (PDF)**. Click here for file
