# Supplementary material for: An integrated approach to epitope analysis II: A system for proteomic-scale prediction of immunological characteristics
Source: Immunome Res. 2010 Nov 2;6:8. doi: 10.1186/1745-7580-6-8 (PMC2991286; doi:10.1186/1745-7580-6-8)
Supplement: Additional File 2 — AntiJen data (PDF). S2a. Table of proteins in AntiJen set. S2b. Summary table of analytical results. S2c. Representative graphics from AntiJen set. [file 1745-7580-6-8-S2.PDF]

Additional Table S2a. Antijen Data Set

As downloaded from http://www.cbs.dtu.dk/suppl/immunology/Bepipred.php

Protein Curation	Amino acids
AAO62007 Mycobacterium_tuberculosis_6_kDa_early_secretory_antigenic_target_(ESAT-6)	95
AAQ55744 Drosophila_melanogaster_DNA_directed_RNA_polymerase_II_largest-subunit	200
ADHE_ECOLI P17547 Aldehyde-alcohol dehydrogenase [Includes: Alcohol dehydrogenase (EC 1.1.1.1) (ADH); Acetaldehyde dehydrogenase [acetylating] (EC 1.2.1.10) (ACDH); Pyruvate-formate-lyase deactivase (PFL deactivase)]. - Escherichia coli, and - Escherich	890
ALL2_ASPFU P79017 Major allergen Asp f 2 precursor (Asp f II). - Aspergillus fumigatus (Sartorya fumigata).	310
APOA4_HUMAN P06727 Apolipoprotein A-IV precursor (Apo-AIV) (ApoA-IV). - Homo sapiens (Human).	396
CAC1A_HUMAN O00555 Voltage-dependent P/Q-type calcium channel alpha-1A subunit (Voltage-gated calcium channel alpha subunit Cav2.1) (Calcium channel, L type, alpha-1 polypeptide isoform 4) (Brain calcium channel I) (BI). - Homo sapiens (Human).	2505
CAF1_YERPE P26948 F1 capsule antigen precursor. - Yersinia pestis.	170
CARP_CANTR Q00663 Candidapepsin precursor (EC 3.4.23.24) (Aspartate protease) (ACP). - Candida tropicalis (Yeast).	394
CARP2_CANAL P28871 Candidapepsin 2 precursor (EC 3.4.23.24) (Aspartate protease 2) (ACP 2) (Secreted aspartic protease 2). - Candida albicans (Yeast).	398
CH10_MYCTU P09621 10 kDa chaperonin (Protein Cpn10) (groES protein) (BCG-A heat shock protein) (10 kDa antigen). - Mycobacterium tuberculosis.	99
CO1A1_HUMAN P02452 Collagen alpha 1(I) chain precursor. - Homo sapiens (Human).	1464
CO2A1_HUMAN P02458 Collagen alpha 1(II) chain precursor [Contains: Chondrocalcin]. - Homo sapiens (Human).	1418
CO2A1_MOUSE P28481 Collagen alpha 1(II) chain precursor [Contains: Chondrocalcin]. - Mus musculus (Mouse).	1459
COA3_AAV2 P03135 Probable coat protein 3. - Adeno-associated virus 2 (AAV2).	504
COAT_FCVC6 P27404 Capsid protein precursor (Coat protein). - Feline calicivirus (strain CFI/68 FIV) (FCV).	668
CORA_HPBVY P03146 Core antigen. - Hepatitis B virus (subtype ayw).	183
CPXA_PSEPU P00183 Cytochrome P450-cam (EC 1.14.15.1) (Camphor 5-monooxygenase) (P450cam). - Pseudomonas putida.	414
DNAK_CHLTR P17821 Chaperone protein dnaK (Heat shock protein 70) (Heat shock 70 kDa protein) (HSP70) (75 kDa membrane protein). - Chlamydia trachomatis.	659
EBN1_EBV P03211 Epstein-Barr nuclear antigen-1 (EBNA-1). - Epstein-Barr virus (strain B95-8) (HHV-4) (Human herpesvirus 4).	641
ENV_CAEVG P31627 Env polyprotein precursor (Coat polyprotein) [Contains: Surface protein; Transmembrane protein]. - Caprine arthritis encephalitis virus (strain G63) (CAEV).	942
ETXB_STAAU P01552 Enterotoxin type B precursor (SEB). - Staphylococcus aureus.	266
FLA1_BORBU P11089 Flagellar filament 41 kDa core protein (Flagellin) (P41) (41 kDa antigen). - Borrelia burgdorferi (Lyme disease spirochete).	336
FMC1_ECOLI P02971 CFA/I fimbrial subunit B precursor (Colonization factor antigen I subunit B) (CFA/I pilin) (CFA/I antigen). - Escherichia coli.	170
G3P_SCHMA P20287 Glyceraldehyde-3-phosphate dehydrogenase (EC 1.2.1.12) (GAPDH) (Major larval surface antigen) (P-37). - Schistosoma mansoni (Blood fluke).	338
GLPA_HUMAN P02724 Glycophorin A precursor (PAS-2) (Sialoglycoprotein alpha) (MN sialoglycoprotein) (CD235a antigen). - Homo sapiens (Human).	150
GLPC_HUMAN P04921 Glycophorin C (PAS-2') (Glycoprotein beta) (GLPC) (Glycoconnectin) (Sialoglycoprotein D) (Glycophorin D) (GPD). - Homo sapiens (Human).	128

GTFC_STRMU P13470 Glucosyltransferase-SI precursor (EC 2.4.1.5) (GTF-SI) (Dextransucrase) (Sucrose 6-glucosyltransferase). - Streptococcus mutans.	1455
HEMA_MEASH P06830 Hemagglutinin-neuraminidase (EC 3.2.1.18). - Measles virus (strain Halle) (Subacute sclerose panencephalitis - virus).	617
HEMA_MEASY P28081 Hemagglutinin-neuraminidase (EC 3.2.1.18). - Measles virus (strain Yamagata-1) (Subacute sclerose panencephalitis - virus).	620
HEMA_RINDR P41355 Hemagglutinin-neuraminidase (EC 3.2.1.18). - Rinderpest virus (strain RBOK) (RDV).	609
HS70_LEIDO Leishmania_donovani_Heat_Shock_protein_70-kDa	653
K11B_LEIIN Kinetoplastid_membrane_protein-11	92
K2C8_HUMAN P05787 Keratin, type II cytoskeletal 8 (Cytokeratin 8) (K8) (CK 8). - Homo sapiens (Human).	482
LACB_BOVIN P02754 Beta-lactoglobulin precursor (Beta-LG) (Allergen Bos d 5). - Bos taurus (Bovine).	178
MP70_MYCTU P0A668 Immunogenic protein MPT70 precursor. - Mycobacterium tuberculosis.	193
MSA2_PLAFC Q99317 Merozoite surface antigen 2 precursor (MSA-2) (Allelic form 1). - Plasmodium falciparum (isolate Camp / Malaysia).	262
MSP1_PLAFK P04932 Merozoite surface protein 1 precursor (Merozoite surface antigens) (PMMSA) (P190). - Plasmodium falciparum (isolate K1 / Thailand).	1630
NCAP_PUUMS P27313 Nucleocapsid protein (Nucleoprotein). - Puumala virus (strain Sotkamo/V-2969/81).	433
O56652 Adeno_associated_virus_2-VP-2	735
O92917 Adeno_associated_virus_2-VP-3	533
OM1A_CHLTR P23732 Major outer membrane protein, serovar A precursor (MOMP). - Chlamydia trachomatis.	396
OM1B_CHLTR P23421 Major outer membrane protein, serovar B precursor (MOMP). - Chlamydia trachomatis.	394
OM1E_CHLPS P10332 Major outer membrane protein precursor (MOMP). - Chlamydia psittaci (Chlamydophila psittaci).	402
OM1E_CHLTR P17451 Major outer membrane protein, serovar E precursor (MOMP). - Chlamydia trachomatis.	393
OM1L_CHLTR P19542 Major outer membrane protein, serovar L1 precursor (MOMP). - Chlamydia trachomatis.	393
OM1M_CHLTR P06597 Major outer membrane protein, serovar L2 precursor (MOMP). - Chlamydia trachomatis.	394
OM1N_CHLTR P23114 Major outer membrane protein, serovar L3 precursor (MOMP). - Chlamydia trachomatis.	397
OMPA1_NEIMC P13415 Major outer membrane protein P.IA precursor (Protein IA) (PIA) (Class 1 protein). - Neisseria meningitidis (serogroup C).	393
OMPB1_NEIMB P30690 Major outer membrane protein P.IB precursor (Protein IB) (PIB) (Porin) (Class 3 protein). - Neisseria meningitidis (serogroup B).	331
OMPF_ECOLI P02931 Outer membrane protein F precursor (Porin ompF) (Outer membrane protein 1A) (Outer membrane protein IA) (Outer membrane protein B). - Escherichia coli.	362
OS25_PLAFO P13829 25 kDa ookinete surface antigen precursor (Pfs25). - Plasmodium falciparum (isolate NF54).	217
OSPC2_BORBU Q08137 Outer surface protein C precursor (PC). - Borrelia burgdorferi (Lyme disease spirochete).	212
P34_SOYBN Soybean_Gly_Bd_30K	379
PA2A_CRODU P08878 Crotoxin acid chain precursor (CA) (Crotapotin). - Crotalus durissus terrificus (South American rattlesnake).	138
PERT_BORPE P14283 Pertactin precursor (P.93) [Contains: Outer membrane protein P.69]. - Bordetella pertussis.	910
PGS2_BOVIN P21793 Decorin precursor (Bone proteoglycan II) (PG-S2). - Bos taurus (Bovine).	360
POLG_DEN2J P07564 Genome polyprotein [Contains: Capsid protein C (Core protein); Envelope	3391

protein M (Matrix protein); Major envelope protein E; Nonstructural protein 1 (NS1); Nonstructural protein 2A (NS2A); Flavivirin protease NS2B regulatory subunit;	
POLG_FMDVO P03305 Genome polyprotein [Contains: Leader protease (EC 3.4.22.46) (P20A); Coat protein VP4; Coat protein VP2; Coat protein VP3; Coat protein VP1; Core protein p12; Core protein p34; Core protein p14; Genome- linked protein VPG; Protease (EC	2332
POLG_HCV1 P26664 Genome polyprotein [Contains: Capsid protein C (Core protein) (p21); Envelope glycoprotein E1 (gp32) (gp35); Envelope glycoprotein E2 (gp68) (gp70) (NS1); p7; Protease NS2 (EC 3.4.22.-) (p23) (NS2-3 proteinase); Protease/helicase NS3 (	3011
POLG_HCVBK P26663 Genome polyprotein [Contains: Capsid protein C (Core protein) (p21); Envelope glycoprotein E1 (gp32) (gp35); Envelope glycoprotein E2 (gp68) (gp70) (NS1); p7; Protease NS2 (EC 3.4.22.-) (p23) (NS2-3 proteinase); Protease/helicase NS3 (	3010
PORF_PSEAE P13794 Outer membrane porin F precursor. - Pseudomonas aeruginosa.	350
Q25763 Plasmodium_falciparum_RAP-1	153
Q25784 Plasmodium_falciparum_Merozite_surface_antigen	149
Q26003 Plasmodium_falciparum_Rhoptry_Protein_RAP-1	171
Q26020 Plasmodium_falciparum_Thrombospondin_related_anonymous_protein_(TRAP)	574
Q47105 Escherichia_coli_Nonfimbrial_adhesin_CS31A	278
Q51189 Neisseria_meningitidis_P64k	593
Q80883 Human_papillomavirus_type_16_E6_protein	90
Q81005 Human_papillomavirus_type_16_Major_capsid_protein_L1	494
Q8B5P5 Human_papillomavirus_type_16_E7_protein	77
Q8QQW1 Grapevine_virus_A_capsid_protein	198
Q8UZC2 Dengue_virus_type_2_E_Protein	488
Q93P53 Chlamydia_trachomatis_Major_outer_membrane_protein,_serovar_C	397
Q9JNQ0 Group_A_M1_Streptococcus_inhibitor_of_complement(Sic)_extracellular_protein	274
Q9L8G3 Mycoplasma_agalactiae_AvgC_(30-37)	238
Q9NGD0 Leishmania_infantum_GRP94	771
RAC3_MOUSE P60764 Ras-related C3 botulinum toxin substrate 3 (p21-Rac3). - Mus musculus (Mouse).	192
RAP2B_RAT P61227 Ras-related protein Rap-2b. - Rattus norvegicus (Rat).	183
RASN_HUMAN P01111 Transforming protein N-Ras. - Homo sapiens (Human).	189
REF_HEVBR P15252 Rubber elongation factor protein (REF) (Allergen Hev b 1). - Hevea brasiliensis (Para rubber tree).	137
RHO2_YEAST P06781 RHO2 protein. - Saccharomyces cerevisiae (Baker's yeast).	192
RHOA_HUMAN P61586 Transforming protein RhoA (H12). - Homo sapiens (Human).	193
RHOQ_HUMAN P17081 Rho-related GTP-binding protein RhoQ (Ras-related GTP-binding protein TC10). - Homo sapiens (Human).	205
RNMG_ASPRE P67876 Ribonuclease mitogillin precursor (EC 3.1.27.-) (Restrictocin). - Aspergillus restrictus.	176
RRAS2_MOUSE P62071 Ras-related protein R-Ras2. - Mus musculus (Mouse).	204
RSR1_YEAST P13856 Ras-related protein RSR1. - Saccharomyces cerevisiae (Baker's yeast).	272
SBP_CRYJA Japanese_Cedar_Pollen_Major_Allergen_(Cry_j_1)	374
SPM1_MAGGR P58371 Subtilisin-like proteinase Spm1 precursor (EC 3.4.21.-) (Serine protease of Magnaporthe 1). - Magnaporthe grisea (Rice blast fungus) (Pyricularia grisea).	536
SRPP_HEVBR O82803 Small rubber particle protein (SRPP) (22 kDa rubber particle protein) (22 kDa RPP) (Latex allergen Hev b 3) (27 kDa natural rubber allergen). - Hevea brasiliensis (Para rubber tree).	204
TALDO_HUMAN P37837 Transaldolase (EC 2.2.1.2). - Homo sapiens (Human).	337
TNNI3_HUMAN P19429 Troponin I, cardiac muscle (Cardiac troponin I). - Homo sapiens (Human).	209
TOP1_HUMAN P11387 DNA topoisomerase I (EC 5.99.1.2). - Homo sapiens (Human).	765
TPM_PANST O61379 Tropomyosin (Allergen Pan s 1) (Pan s I). - Panulirus stimpsoni (Spiny	274

lobster).	
TRPB_ECO57 Q8X7B6 Tryptophan synthase beta chain (EC 4.2.1.20). - Escherichia coli O157:H7.	396
UMUD_ECOLI P04153 UmuD protein (EC 3.4.21.-) [Contains: UmuD' protein]. - Escherichia coli, - Escherichia coli O157:H7, and - Shigella flexneri.	139
URE2_HELPY P69996 Urease beta subunit (EC 3.5.1.5) (Urea amidohydrolase). - Helicobacter pylori (Campylobacter pylori).	569
VCO7_ADE05 P68951 Major core protein precursor (Protein VII) (pVII). - Human adenovirus 5 (HAdV-5).	198
VE1_HPV16 P03114 Replication protein E1. - Human papillomavirus type 16.	649
VE2_HPV16 P03120 Regulatory protein E2. - Human papillomavirus type 16.	365
VE6_HPV16 P03126 E6 protein. - Human papillomavirus type 16.	158
VGLB_BHV1C P12640 Glycoprotein I precursor (Glycoprotein GVP-6) (Glycoprotein 11A) (Glycoprotein 16) (Glycoprotein G130) (Glycoprotein B). - Bovine herpesvirus 1.1 (strain Cooper) (BoHV-1) (Infectious bovine - rhinotracheitis virus).	932
VGLD_CHV1 P36342 Glycoprotein D precursor. - Cercopithecine herpesvirus 1 (CeHV-1) (Simian herpes B virus).	395
VGLE_VZVD P09259 Glycoprotein E precursor (Glycoprotein GI). - Varicella-zoster virus (strain Dumas) (VZV).	623
VGLG_HHV11 P06484 Glycoprotein G. - Human herpesvirus 1 (strain 17) (HHV-1) (Human herpes simplex virus - 1).	238
VGLG_HHV2H P13290 Glycoprotein G. - Human herpesvirus 2 (strain HG52) (HHV-2) (Human herpes simplex virus - 2).	699
VL1_BPV1 P03103 Major capsid protein L1. - Bovine papillomavirus type 1.	495
VL1_BPV2 P06458 Major capsid protein L1. - Bovine papillomavirus type 2.	497
VL1_CRPVK P03102 Major capsid protein L1. - Cottontail rabbit (shope) papillomavirus (strain Kansas) (CRPV).	505
VL1_HPV11 P04012 Major capsid protein L1. - Human papillomavirus type 11.	501
VL1_HPV16 P03101 Major capsid protein L1. - Human papillomavirus type 16.	531
VL1_HPV18 P06794 Major capsid protein L1. - Human papillomavirus type 18.	568
VL1_HPV1A P03099 Major capsid protein L1. - Human papillomavirus type 1a.	508
VL1_HPV2A P25486 Major capsid protein L1. - Human papillomavirus type 2a.	510
VL1_HPV31 P17388 Major capsid protein L1. - Human papillomavirus type 31.	504
VL1_HPV6B P69899 Major capsid protein L1. - Human papillomavirus type 6b.	500
VL2_BPV4 P08342 Minor capsid protein L2. - Bovine papillomavirus type 4.	357
VL2_HPV11 P04013 Minor capsid protein L2. - Human papillomavirus type 11.	455
VL2_HPV16 P03107 Minor capsid protein L2. - Human papillomavirus type 16.	473
VL2_HPV1A P03105 Minor capsid protein L2. - Human papillomavirus type 1a.	507
VL2_HPV6B P03106 Minor capsid protein L2. - Human papillomavirus type 6b.	459
VMSA_HPBV9 P17101 Major surface antigen precursor. - Hepatitis B virus (subtype adw / strain 991).	400
VMSA_HPBVA P24025 Major surface antigen precursor. - Hepatitis B virus (strain alpha1).	389
VP2_AHSV4 P32553 Outer capsid protein VP2. - African horse sickness virus 4 (AHSV-4) (African horse sickness virus - (serotype 4)).	1060
YPX1_BLVJ P03412 Hypothetical PXBL-I protein (Fragment). - Bovine leukemia virus (Japanese isolate BLV-1) (BLV).	308

Additional Table S2b. B-cell Epitopes defined in Antijen data set and predicted B-cell epitopes and MHC high affinity binding peptides predicted.

Summary statistics from analyses of 124 proteins derived from mammalian, viral, bacterial, protozoal, reptilian, plant species. In many cases a B-cell epitope overlapped with more than one MHC high affinity binding region; similarly individual MHC high affinity peptides in some cases comprised more than one B-cell epitope. We have restricted the summary of overlaps to those with at least one overlap. B-cell epitopes were those in the top 25% probability; MHC-I and MHC-II were those within the top 25% predicted binding affinity.

Epitopes within the Antijen dataset are designated as uppercase 'E' and lowercase 'e'; the origins of this coding is not clear (noted by Larsen *et al*). We elected to use a binary coding system combining both upper and lower case ‘E” as the experimental mapping.

Total proteins in Antijen Data set		124	
Total B cell epitopes mapped in Antijen		243	
Mapped in Integrated System			
B cell epitopes >4aa long	2277	Total B-cell epitopes overlapping or borders within 3 aa of a MHC-I or MHC-II high affinity binding peptide	468 at least one MHC-I<br>332 at least one MHC-II
MHC-I High affinity binding regions	1005	MHC-I high affinity binding peptides which overlap or bordering within 3 aa of a high probability B cell epitope	792 (at least one B-cell epitope)
MHC-II High affinity binding regions	717	Percentage of MHC-II high affinity binding peptides which overlap or bordering within 3 aa of a high probability B cell epitope	688 (at least one B-cell epitope)
		MHC-I overlapping MHC-II high affinity binding peptides	500 (at least one MHC overlap)

Additional Figures S2c: Representative graphics from Antijen

Graphics have been generated for all proteins in Antijen. To keep within file size restrictions a representative subset of plots for 15 representative proteins is provided here.
Lines and ribbons are as described in Figure 5. In addition the B-cell epitope mapping provided in Antijen is shown as a red horizontal ribbon. Where additional literature references recorded B-cell epitope maps these are superimposed in green.

Overlay Plot

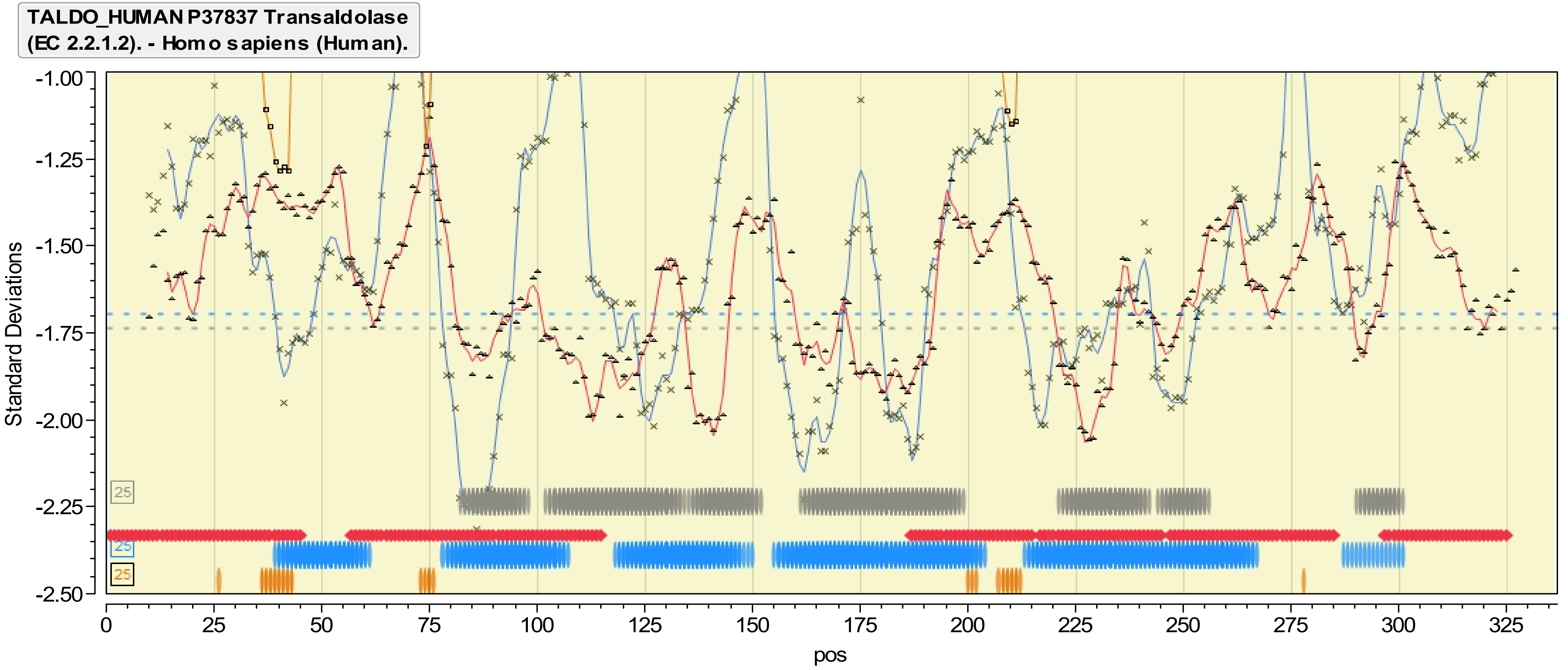

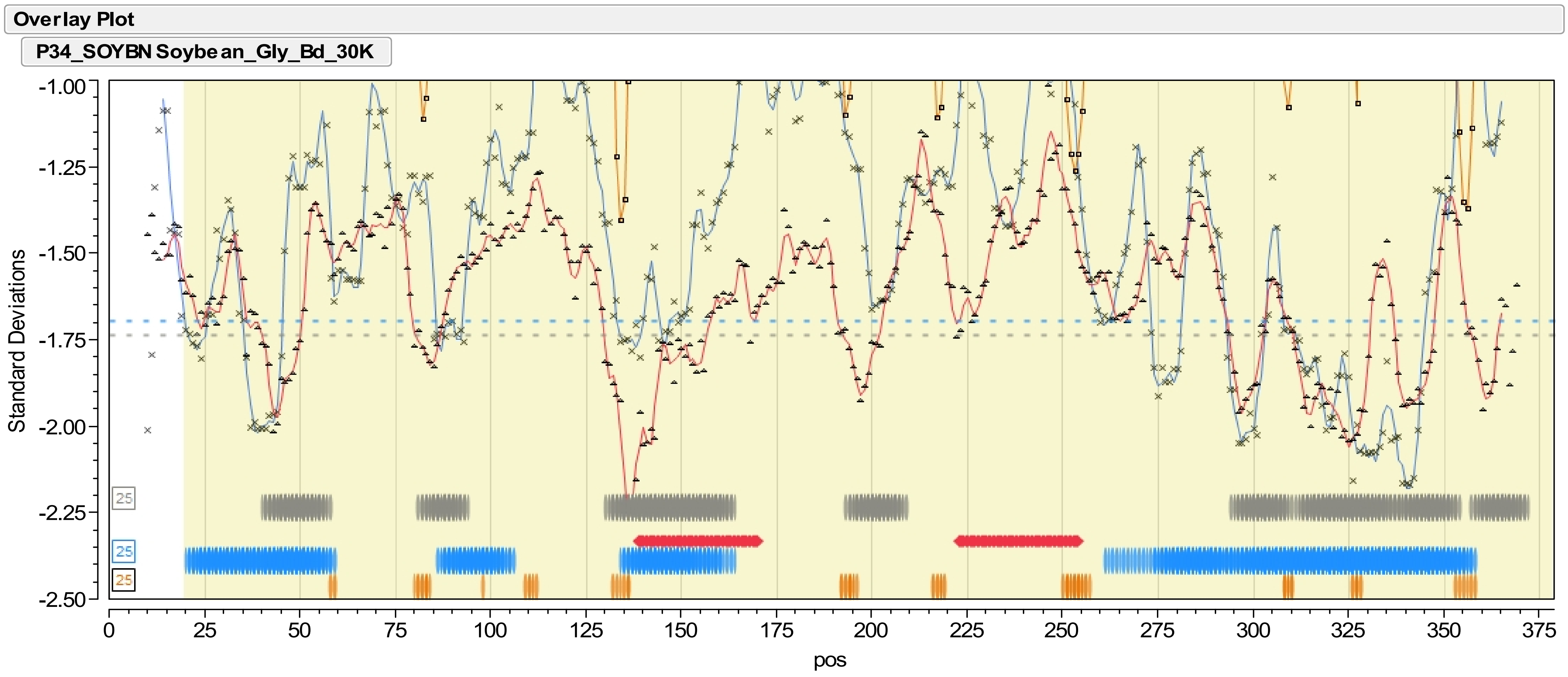
Overlay Plot
P34_SOYBN Soybean_Gly_Bd_30K
Standard Deviations
pos
-1.00
-1.25
-1.50
-1.75
-2.00
-2.25
-2.50
0
25
50
75
100
125
150
175
200
225
250
275
300
325
350
375
25
25
25

Overlay Plot

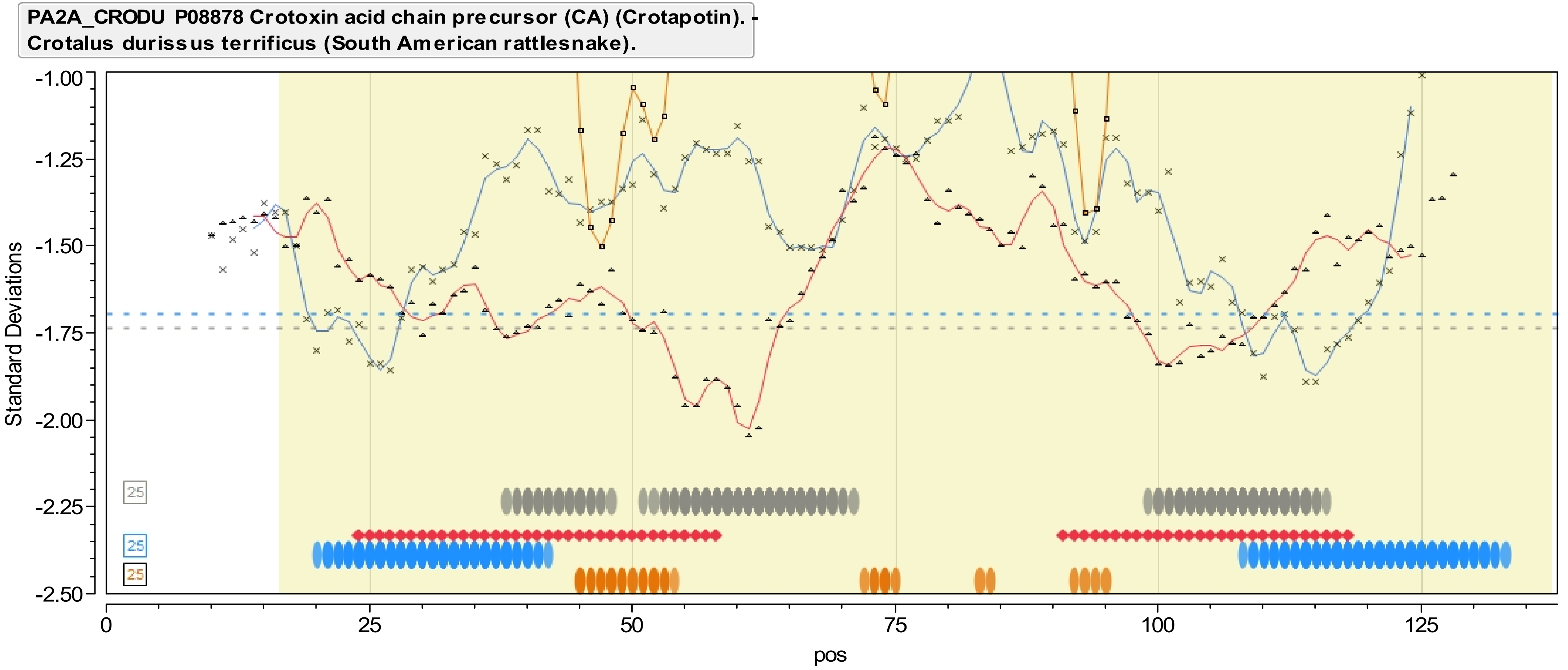

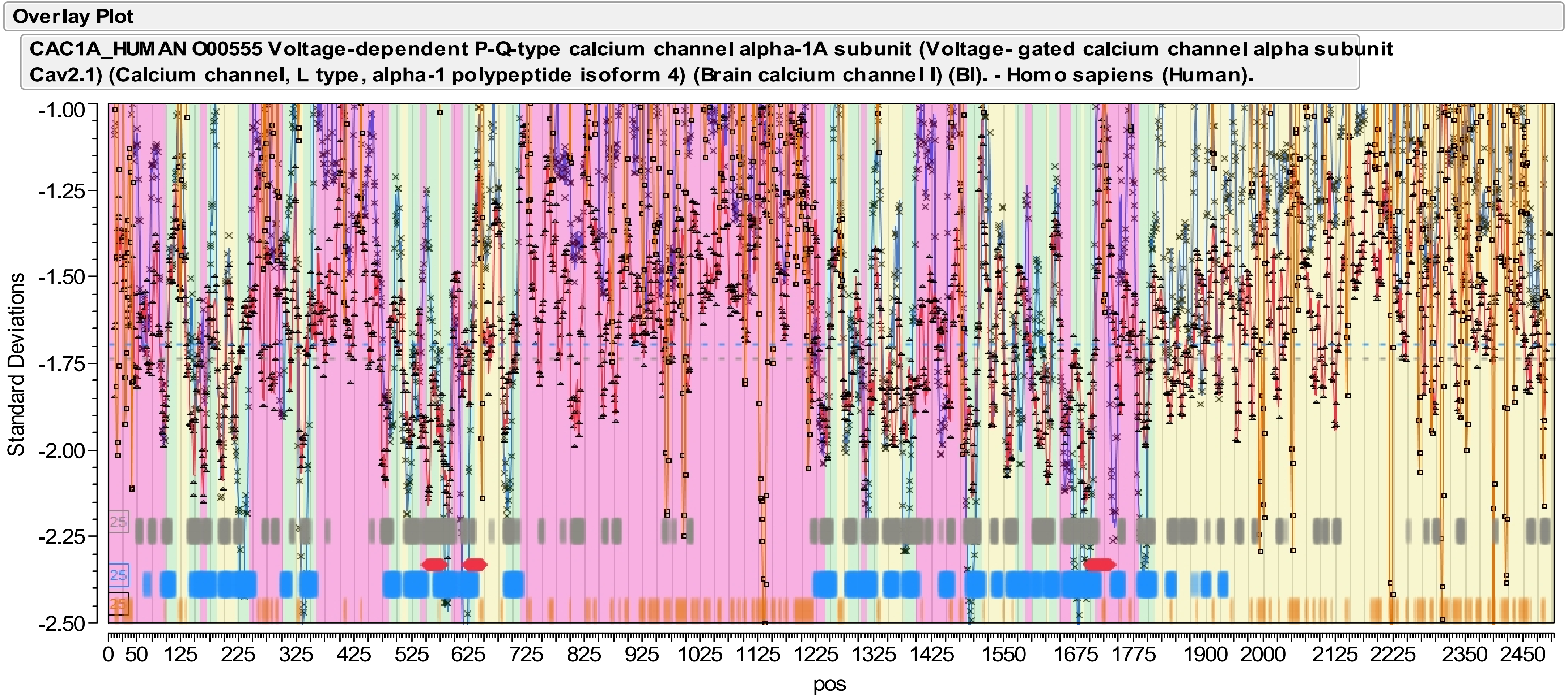
Overlay Plot
CAC1A_HUMAN O00555 Voltage-dependent P-Q-type calcium channel alpha-1A subunit (Voltage- gated calcium channel alpha subunit Cav2.1) (Calcium channel, L type, alpha-1 polypeptide isoform 4) (Brain calcium channel I) (BI). - Homo sapiens (Human).
Standard Deviations
-1.00
-1.25
-1.50
-1.75
-2.00
-2.25
-2.50
pos
0 50 125 225 325 425 525 625 725 825 925 1025 1125 1225 1325 1425 1550 1675 1775 1900 2000 2125 2225 2350 2450

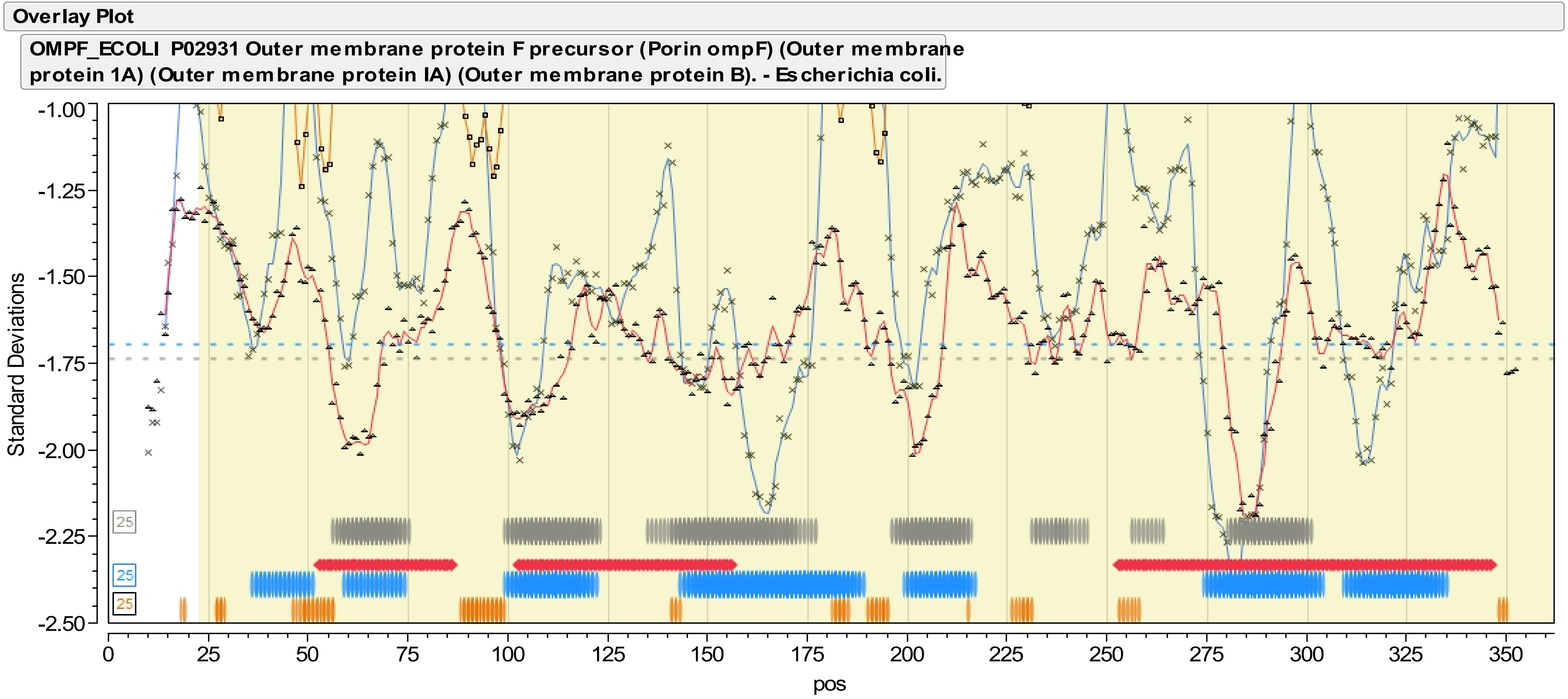

Overlay Plot
OMPF_ECOLI P02931 Outer membrane protein F precursor (Porin ompF) (Outer membrane protein 1A) (Outer membrane protein IA) (Outer membrane protein B). - Escherichia coli.
Standard Deviations
-1.00
-1.25
-1.50
-1.75
-2.00
-2.25
-2.50
25
25
25
0
25
50
75
100
125
150
175
200
225
250
275
300
325
350
pos

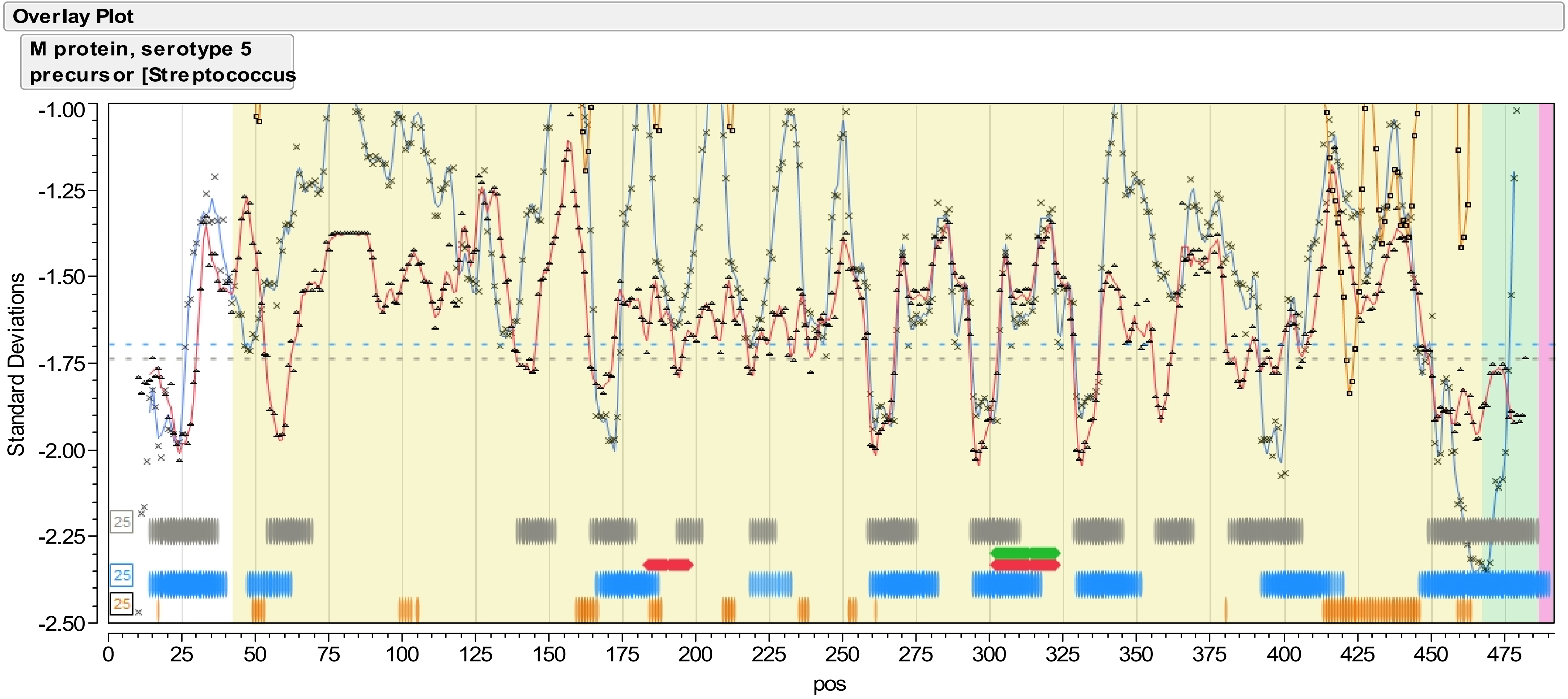
Overlay Plot
M protein, serotype 5
precursor [Streptococcus
-1.00
-1.25
-1.50
-1.75
-2.00
-2.25
-2.50
Standard Deviations
25
25
25
0
25
50
75
100
125
150
175
200
225
250
275
300
325
350
375
400
425
450
475
pos

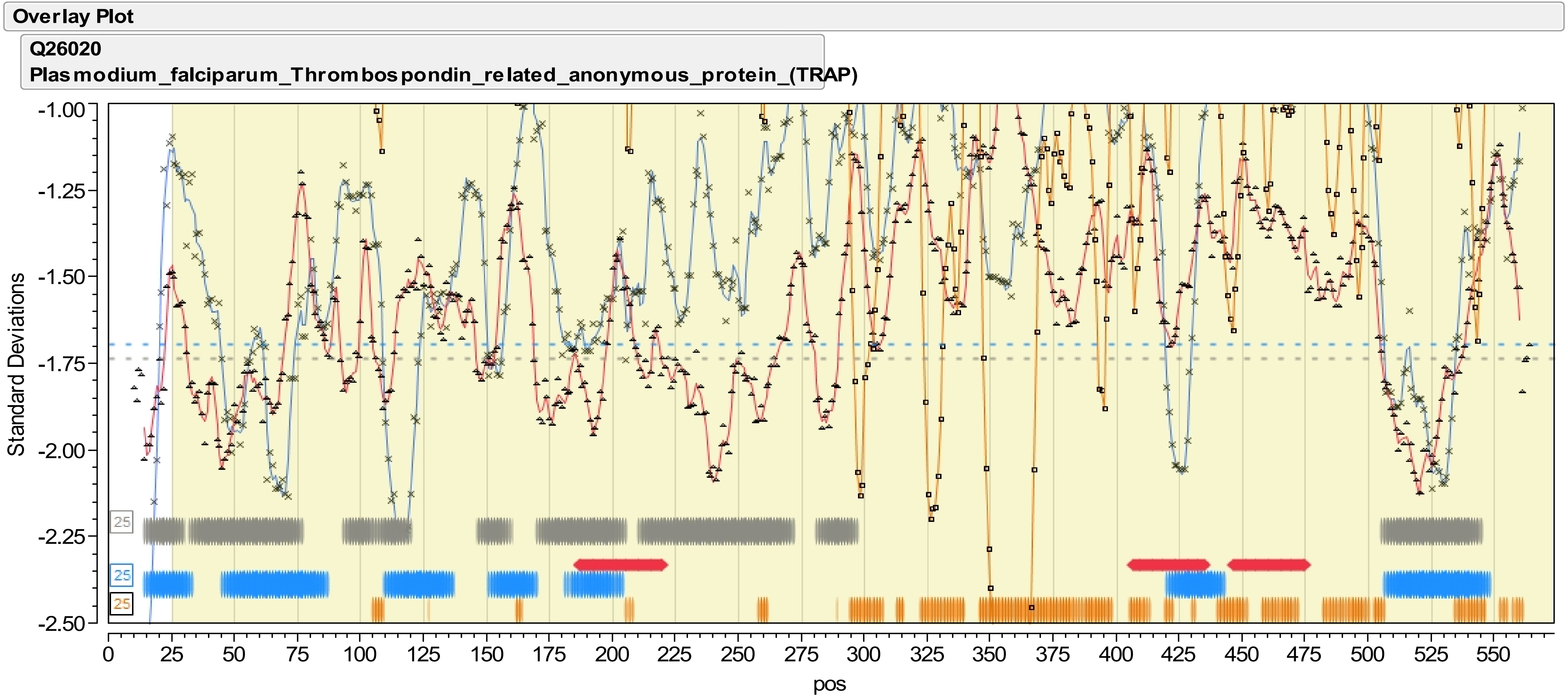

Overlay Plot
Q26020
Plasmodium_falciparum_Thrombospondin_related_anonymous_protein_(TRAP)
Standard Deviations
-1.00
-1.25
-1.50
-1.75
-2.00
-2.25
-2.50
25
25
25
0
25
50
75
100
125
150
175
200
225
250
275
300
325
350
375
400
425
450
475
500
525
550
pos

Overlay Plot

SPM1_MAGGR P58371 Subtilisin-like proteinase Spm1 precursor (EC 3.4.21.-) (Serine protease of Magnaporthe 1). - Magnaporthe grisea (Rice blast fungus) (Pyricularia grisea).

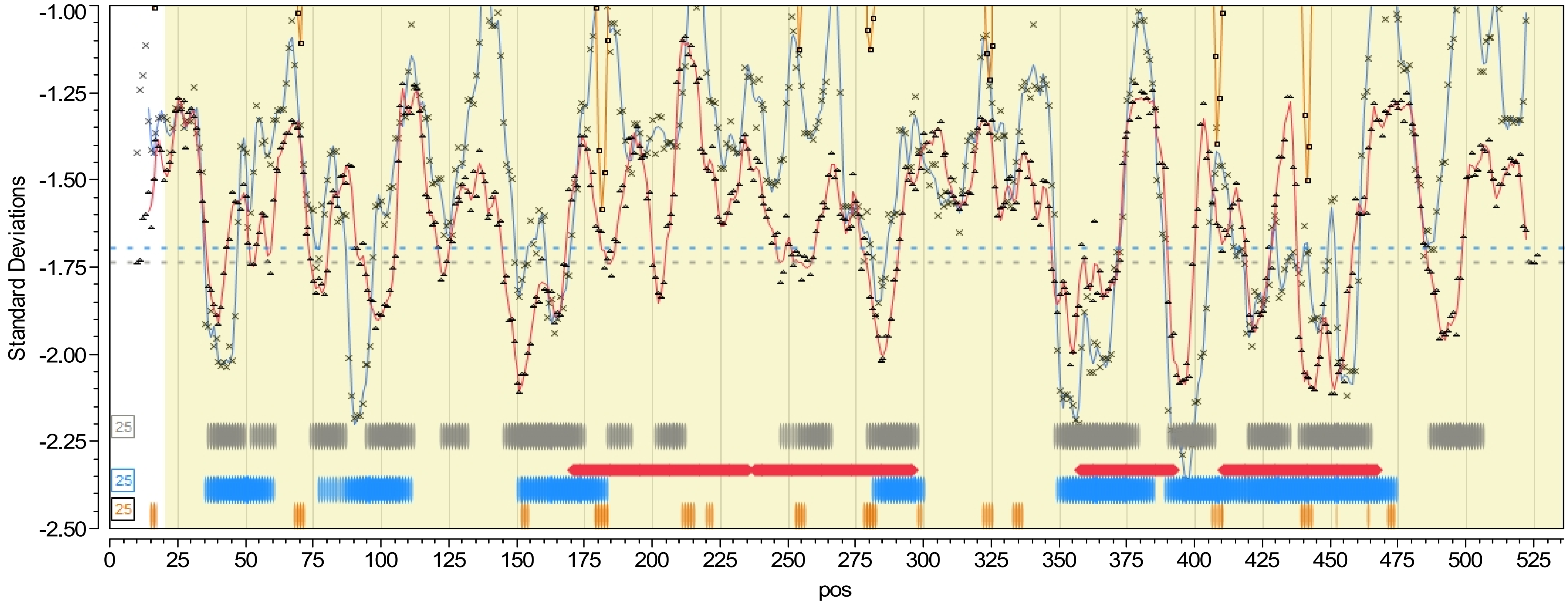

Overlay Plot

EBN1_EBV P03211 Epstein-Barr nuclear antigen-1 (EBNA-1). - Epstein-Barr virus (strain B95-8) (HHV-4) (Human herpesvirus 4).

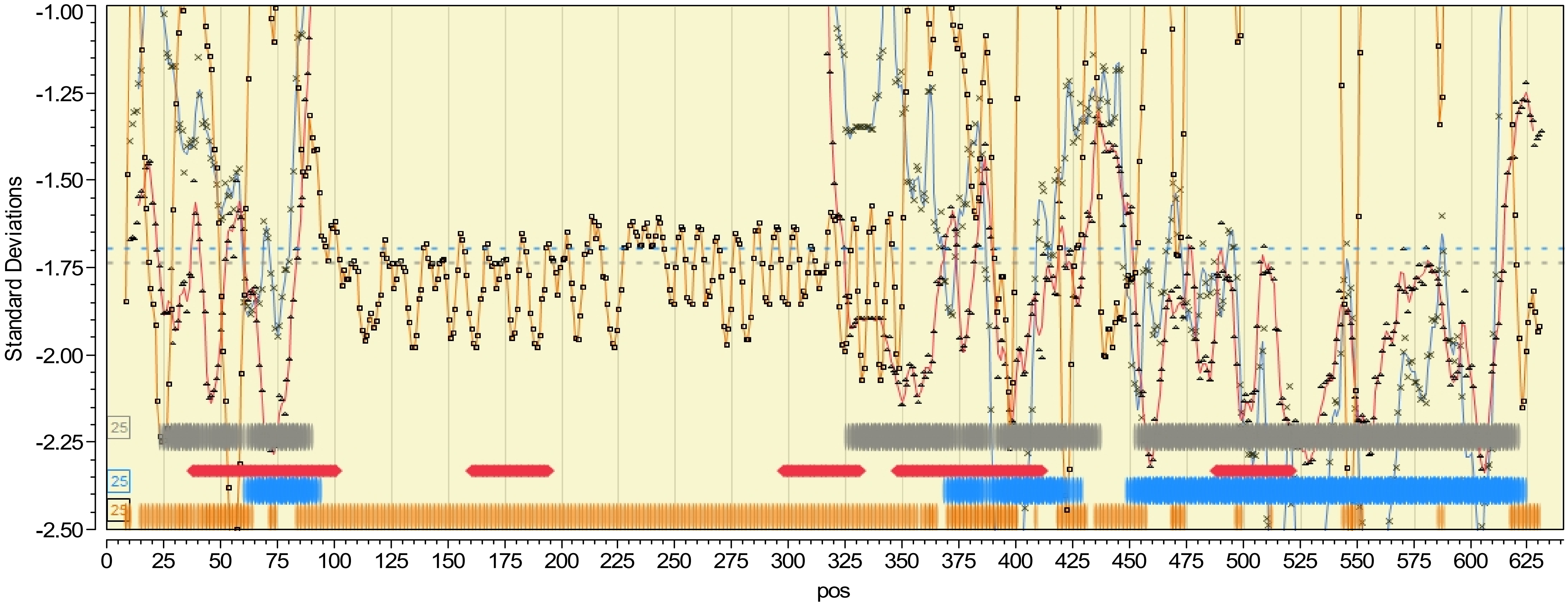

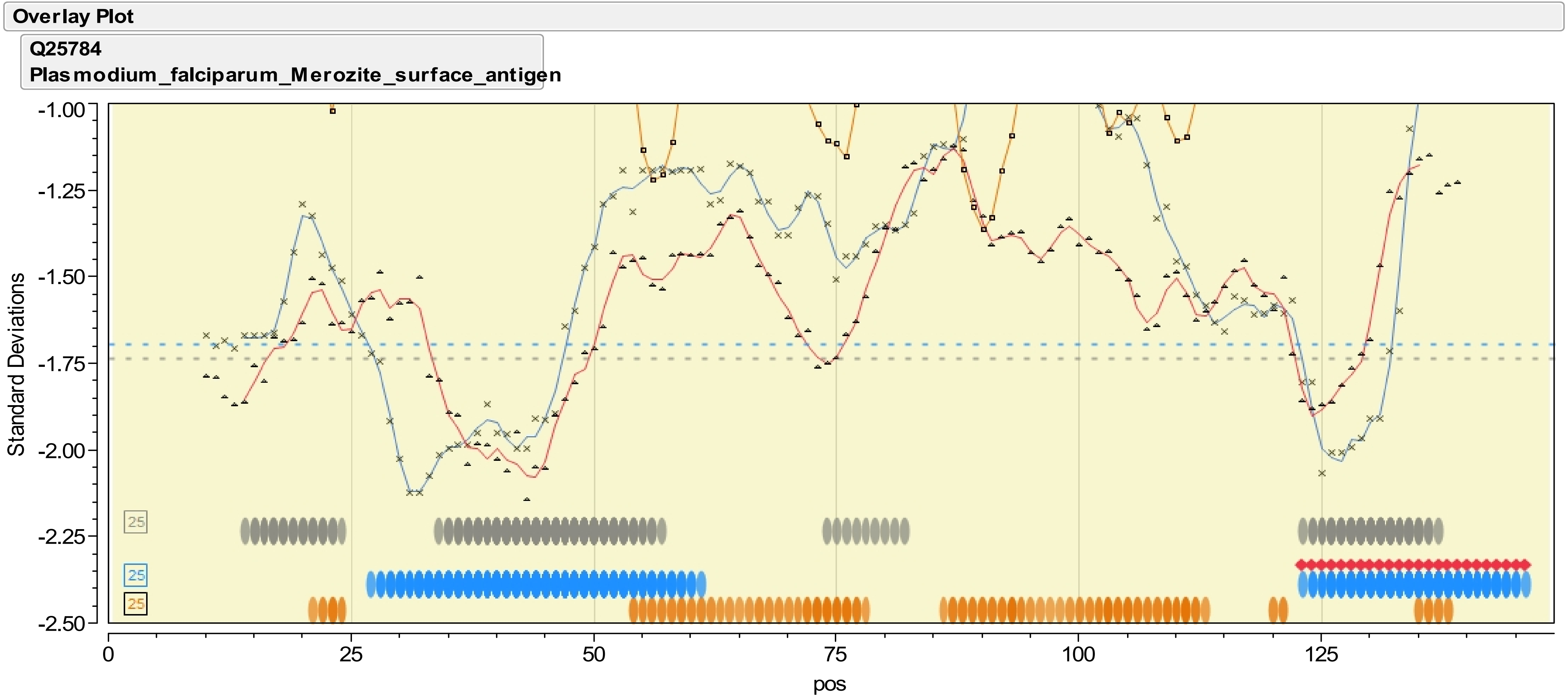

Overlay Plot
Q25784
Plasmodium_falciparum_Merozite_surface_antigen
Standard Deviations
-1.00
-1.25
-1.50
-1.75
-2.00
-2.25
-2.50
25
25
25
0
25
50
75
100
125
pos

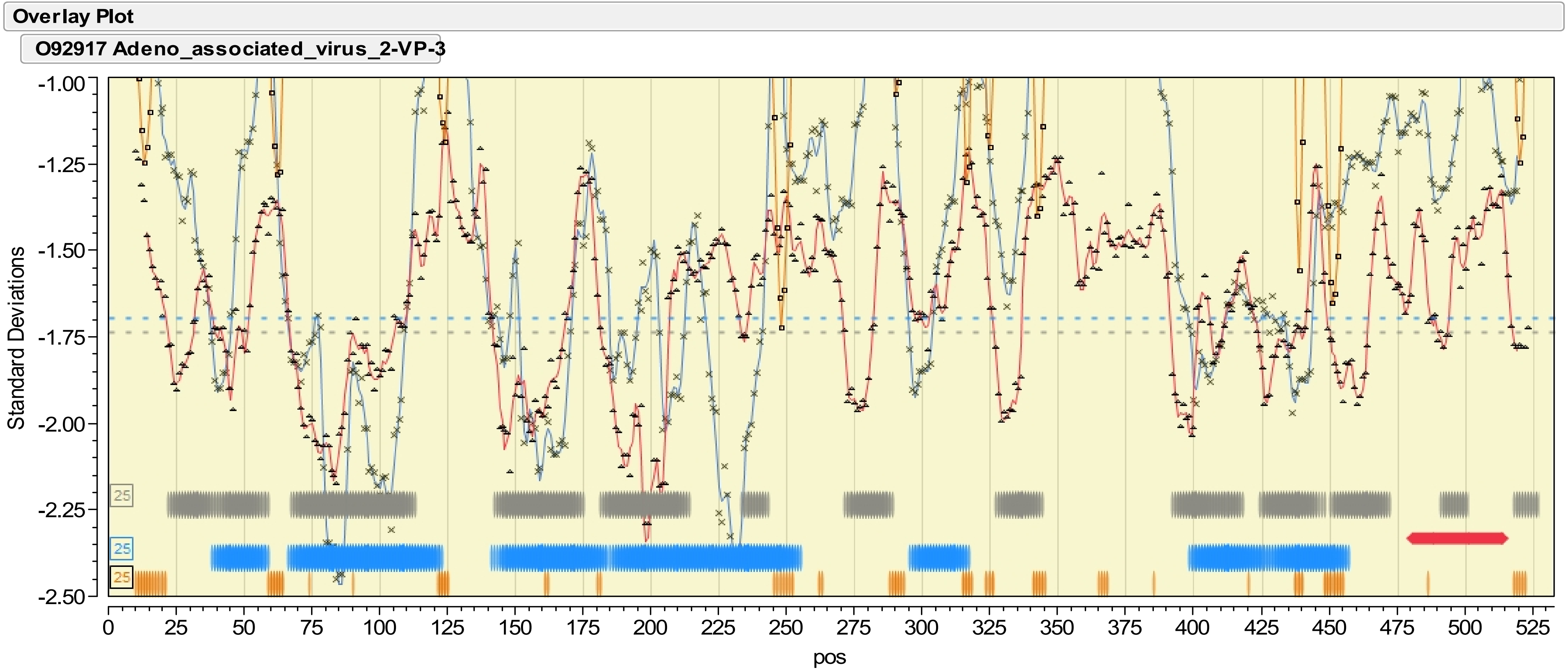

Overlay Plot
O92917 Adeno_associated_virus_2-VP-3
Standard Deviations
-1.00
-1.25
-1.50
-1.75
-2.00
-2.25
-2.50
25
25
25
0
25
50
75
100
125
150
175
200
225
250
275
300
325
350
375
400
425
450
475
500
525
pos

Overlay Plot

ENV_CAEVG P31627 Env polyprotein precursor (Coat polyprotein) [Contains- Surface protein; Transmembrane protein]. - Caprine arthritis encephalitis virus (strain G63) (CAEV).

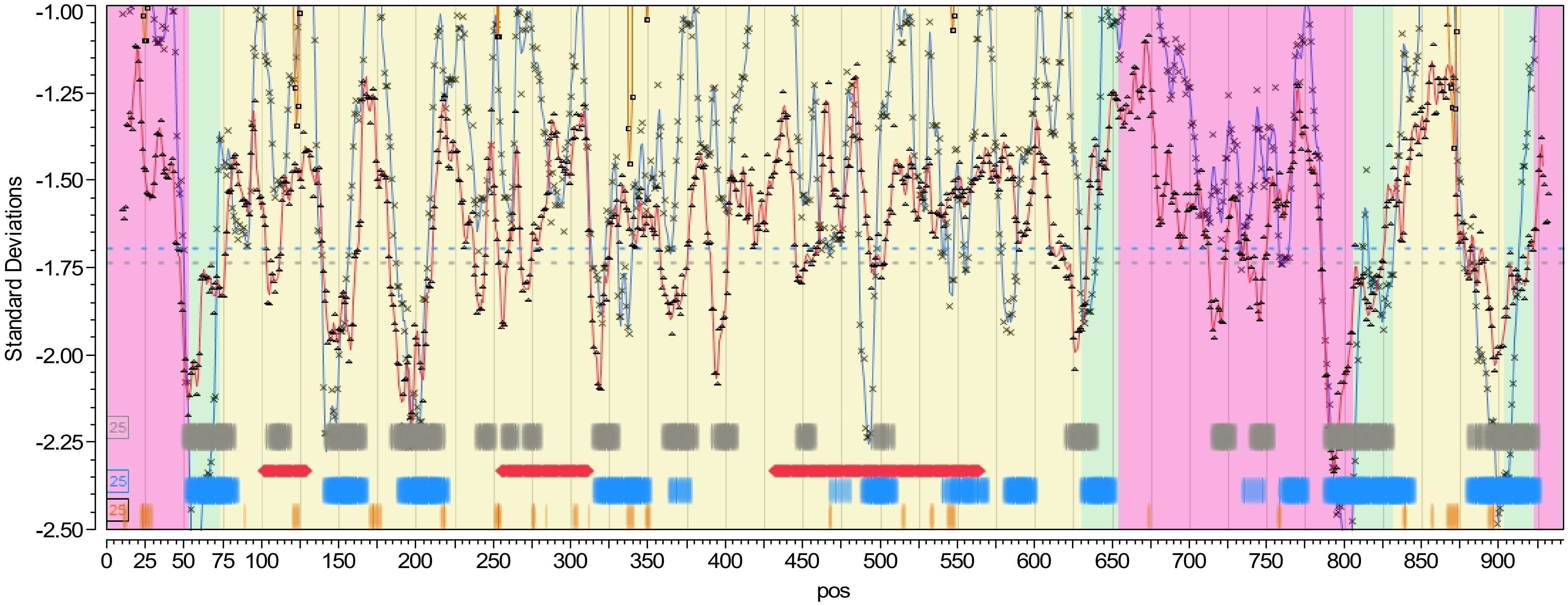

Overlay Plot

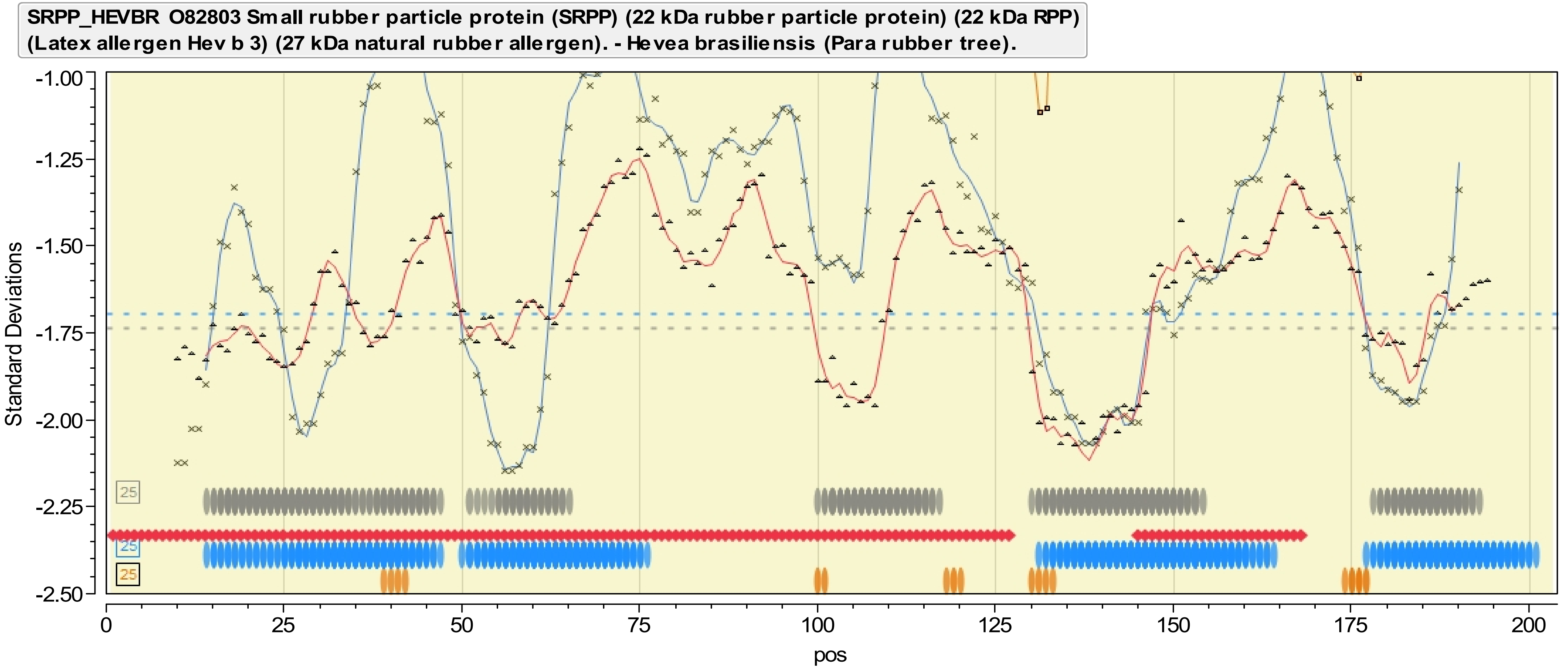

Overlay Plot

Q9JNQ0
Group_A_M1_Streptococcus_inhibitor_of_complement(Sic)_extracellular_protein

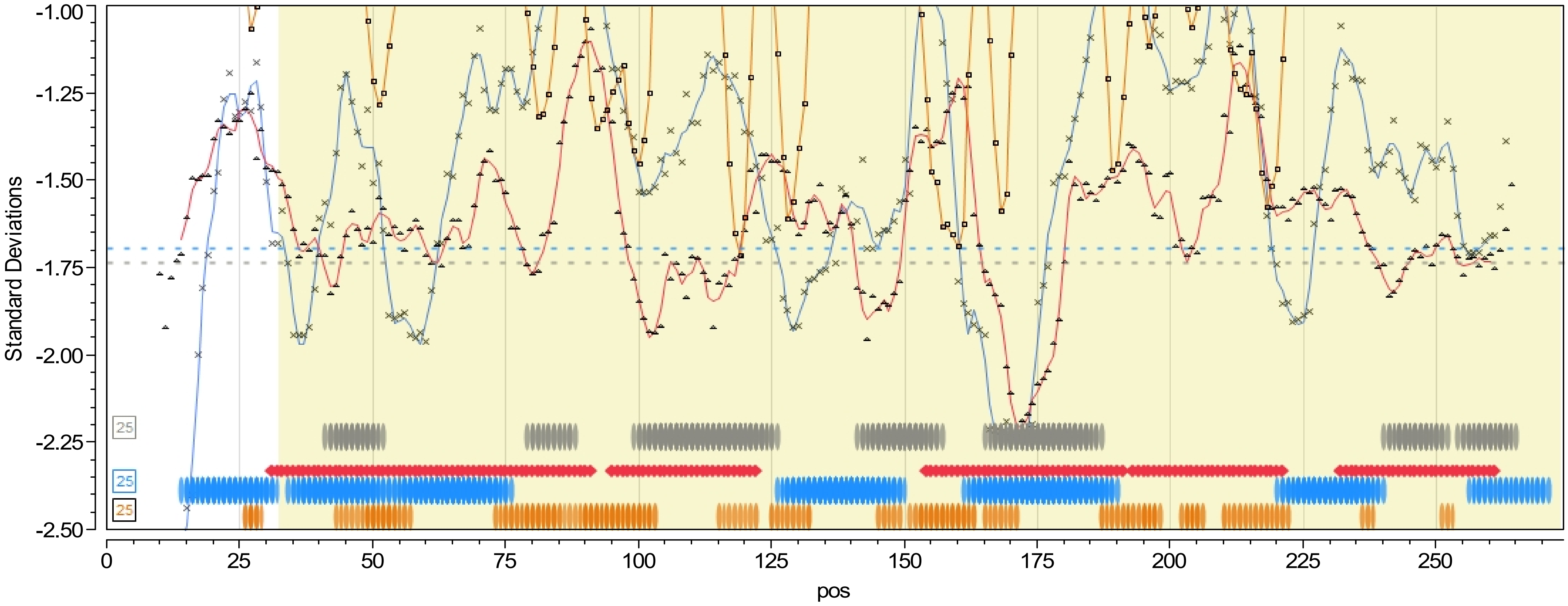

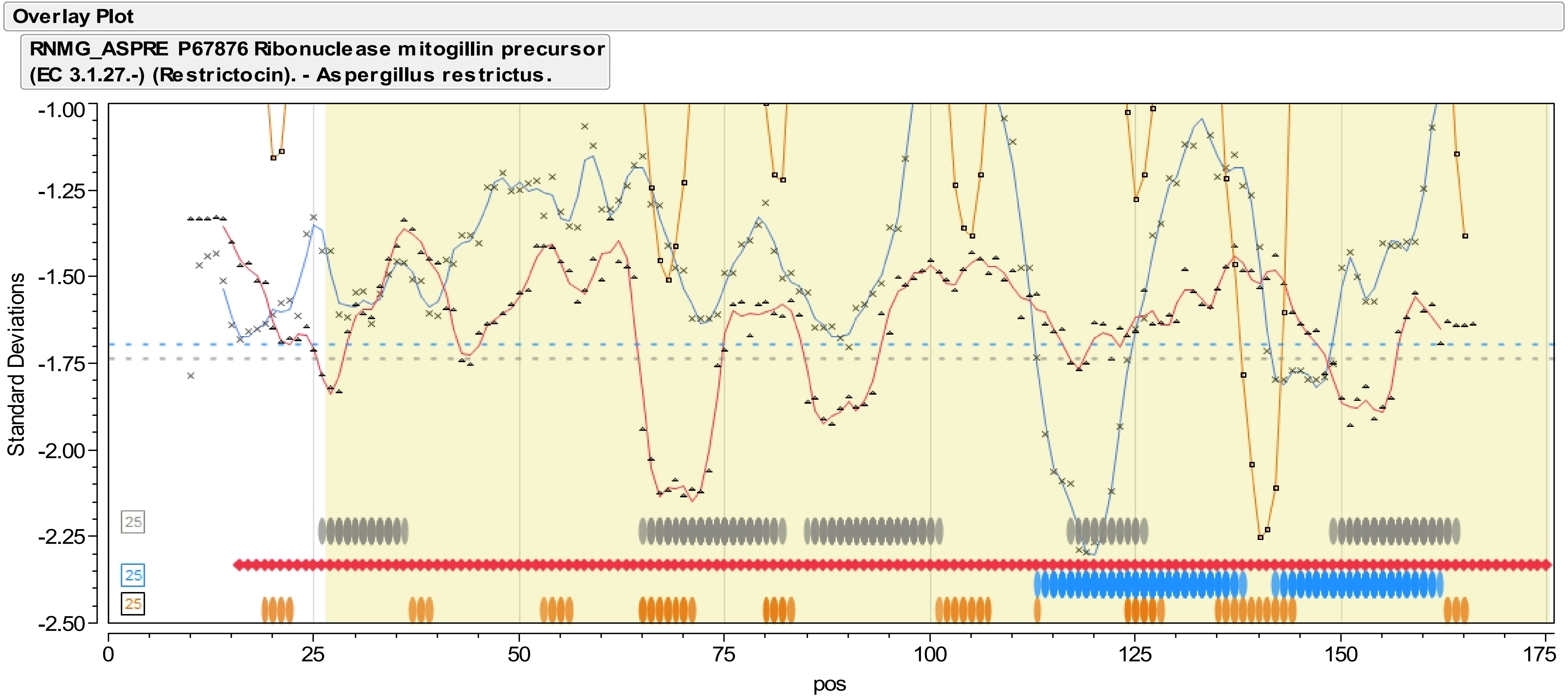

Overlay Plot
RNMG_ASPRE P67876 Ribonuclease mitogillin precursor (EC 3.1.27.-) (Restrictocin). - Aspergillus restrictus.
Standard Deviations
-1.00
-1.25
-1.50
-1.75
-2.00
-2.25
-2.50
25
25
25
0
25
50
75
100
125
150
175
pos
