# Supplementary material for: An integrated approach to epitope analysis II: A system for proteomic-scale prediction of immunological characteristics
Source: Immunome Res. 2010 Nov 2;6:8. doi: 10.1186/1745-7580-6-8 (PMC2991286; doi:10.1186/1745-7580-6-8)
Supplement: Additional File 3 — Staph. aureus data (PDF). S3a. Table of epitopes mapped experimentally in Staph. aureus. S3b. Strains of Staph aureus analyzed. S3c. Maps of three Staph. aureus toxins. S3d. Map of Staph. aureus Protein A. [file 1745-7580-6-8-S3.PDF]

Additional File Table S3a. Mapping of predicted and experimental epitopes in five *Staph. aureus* proteins. Amino acid positions of experimentally defined epitopes are shown with both published amino acid positions and amino acid positions adjusted to include signal peptide or other precursor peptides indicated for each protein. Experimental function mapped is summarized in the last column; see published reports for details. Figure 6 and additional figures in this Additional file show the graphical plot of the epitopes.

(see next page)

	Reported position	Position adjusted for signal peptide or other cleavage	Reported function
**Thermonuclease (NC_002951.57650135)**			
Signal peptide or other cleavage point		1-79	
Predicted CEGs		89-130,162-177,183-199	
Schaeffer [1]	61-80, 81-140	140-159,160-178, 190-225	MHC-II (3 murine alleles)
Nikcevich [2]	91-106	170-185	MHC-II (peptide binding murineI- E^k)
Liu [37]	81-100	160-179	MHC-II (murine)
**Enterotoxin TSST-1 (NC_007622.82750121)**			
Signal peptide or other cleavage		1-40	
Predicted CEGs		75-102, 146-177	
Blanco [3] *et al* 1990<br>Bonventre [4, 5] *et al* 1993,1995<br>Cullen [6, 7]1995,1996<br>Deresiewicz [8]*et al* 1994; Earhart [9] *et al* 1998	13-15,132-144	53-55,172-184	MAb blocks TCR binding sites
Kum [10] *et al* 1996	31,32	71,72	Site specific mutagenesis inihibits MHC-II binding
Kum [11] *et al* 2001	51-56	91-96	MAb block MHC-II binding
**Enterotoxin B (NC_002951.57651597)**			
Signal peptide		1-27	
Predicted CEGs		61-89, 104-130,184-240	
Kappler [12]	9-23;41-53,60-61	36-50;68-80	MHC-II binding . Amino-acids 60-61 involved in Vβ interaction (human)
Nishi [13]	225-234	252-261	Antibody binding (human serum pool)
Wood [14]	21-32;93-107;202-217	48-59;120-134;229-244	Polyclonal antibody binding.
**Iron Regulated Determinant B (IsdB) (NC_002951.57651738)**			
Signal peptide		1-40	
Predicted CEGs		101-149,155-238,243-317,355-435,438-505	
Brown [15]	-NA-	a.130, 139, 180, 182, 217, 226, 454<br>b.130, 157–159, 184, 454<br>c.157–159, 184, 267, 400, 425<br>d.130, 157–159, 184, 454<br>e.157–159, 184<br>f.130, 296, 454	Point mutations which disrupt binding of 6 MAb (a-f)
**ABC Transporter ATP-Binding protein (SA00533 NC_002951.5765.1892)**			
Signal peptide		none	
Predicted CEGs	128-212,221-259,289-335,367-436,441-469		
Burnie [16]	211-216;235-238;302-306;357-363;379-385;403-407; 409-424		Antibody binding

Reference List

1. Schaeffer EB, Sette A, Johnson DL, Bekoff MC, Smith JA, Grey HM, Buus S: **Relative contribution of "determinant selection" and "holes in the T-cell repertoire" to T-cell responses**. *Proc Natl Acad Sci U S A* 1989, **86:**4649-4653.

2. Nikcevich KM, Kopielski D, Finnegan A: **The immunodominant region of Staphylococcal nuclease is represented by multiple peptide sequences**. *Cell Immunol* 1996, **172:**254-261.

3. Blanco L, Choi EM, Connolly K, Thompson MR, Bonventre PF: **Mutants of staphylococcal toxic shock syndrome toxin 1: mitogenicity and recognition by a neutralizing monoclonal antibody**. *Infect Immun* 1990, **58:**3020-3028.

4. Bonventre PF, Heeg H, Edwards CK, III, Cullen CM: **A mutation at histidine residue 135 of toxic shock syndrome toxin yields an immunogenic protein with minimal toxicity**. *Infect Immun* 1995, **63:**509-515.

5. Bonventre PF, Heeg H, Cullen C, Lian CJ: **Toxicity of recombinant toxic shock syndrome toxin 1 and mutant toxins produced by Staphylococcus aureus in a rabbit infection model of toxic shock syndrome**. *Infect Immun* 1993, **61:**793-799.

6. Cullen CM, Blanco LR, Bonventre PF, Choi E: **A toxic shock syndrome toxin 1 mutant that defines a functional site critical for T-cell activation**. *Infect Immun* 1995, **63:**2141-2146.

7. Cullen CM, Bonventre PF, Heeg H, Bluethmann H, Mountz JD, Edwards CK, III: **A fas antigen receptor mutation allows development of toxic shock syndrome toxin-1-induced lethal shock in V beta 8.2 T-cell receptor transgenic mice**. *Pathobiology* 1995, **63:**293-304.

8. Deresiewicz RL, Woo J, Chan M, Finberg RW, Kasper DL: **Mutations affecting the activity of toxic shock syndrome toxin-1**. *Biochemistry* 1994, **33:**12844-12851.

9. Earhart CA, Mitchell DT, Murray DL, Pinheiro DM, Matsumura M, Schlievert PM, Ohlendorf DH: **Structures of five mutants of toxic shock syndrome toxin-1 with reduced biological activity**. *Biochemistry* 1998, **37:**7194-7202.

10. Kum WW, Wood JA, Chow AW: **A mutation at glycine residue 31 of toxic shock syndrome toxin-1 defines a functional site critical for major histocompatibility complex class II binding and superantigenic activity**. *J Infect Dis* 1996, **174:**1261-1270.

11. Kum WW, Chow AW: **Inhibition of staphylococcal enterotoxin A-induced superantigenic and lethal activities by a monoclonal antibody to toxic shock syndrome toxin-1**. *J Infect Dis* 2001, **183:**1739-1748.

12. Kappler JW, Herman A, Clements J, Marrack P: **Mutations defining functional regions of the superantigen staphylococcal enterotoxin B**. *J Exp Med* 1992, **175:**387-396.

13. Nishi JI, Kanekura S, Takei S, Kitajima I, Nakajima T, Wahid MR, Masuda K, Yoshinaga M, Maruyama I, Miyata K: **B cell epitope mapping of the bacterial superantigen staphylococcal enterotoxin B: the dominant epitope region recognized by intravenous IgG**. *J Immunol* 1997, **158:**247-254.

14. Wood AC, Chadwick JS, Brehm RS, Todd I, Arbuthnott JP, Tranter HS: **Identification of antigenic sites on staphylococcal enterotoxin B and toxoid**. *FEMS Immunol Med Microbiol* 1997, **17:**1-10.

15. Brown M, Kowalski R, Zorman J, Wang XM, Towne V, Zhao Q, Secore S, Finnefrock AC, Ebert T, Pancari G, Isett K, Zhang Y, Anderson AS, Montgomery D, Cope L, McNeely T: **Selection and characterization of murine monoclonal antibodies to Staphylococcus aureus iron-regulated surface determinant B with functional activity in vitro and in vivo**. *Clin Vaccine Immunol* 2009, **16:**1095-1104.

16. Burnie JP, Matthews RC, Carter T, Beaulieu E, Donohoe M, Chapman C, Williamson P, Hodgetts SJ: **Identification of an immunodominant ABC transporter in methicillin-resistant Staphylococcus aureus infections**. *Infect Immun* 2000, **68:**3200-3209.

Additional File S3a. Strains of *Staphylococcus aureus* analyzed

Genbank Proteome	Strain
NC_002745	Staphylococcus aureus strain N315
NC_002758	Staphylococcus aureus strain Mu50
NC_002951	Staphylococcus aureus strain COL
NC_002952	Staphylococcus aureus strain MRSA252
NC_002953	Staphylococcus aureus strain MSSA476
NC_003923	Staphylococcus aureus strain MW2
NC_007622	Staphylococcus aureus strain RF122
NC_007793	Staphylococcus aureus strain USA300 FPR3757
NC_007795	Staphylococcus aureus strain NCTC8325
NC_009487	Staphylococcus aureus strain JH9
NC_009632	Staphylococcus aureus strain JH1
NC_009641	Staphylococcus aureus strain Newman
NC_009782	Staphylococcus aureus strain Mu3
NC_010063	Staphylococcus aureus strain USA300 TCH1516
NC_013450	Staphylococcus aureus strain ED98

Additional Figures S3c. Three *Staph. aureus* toxins

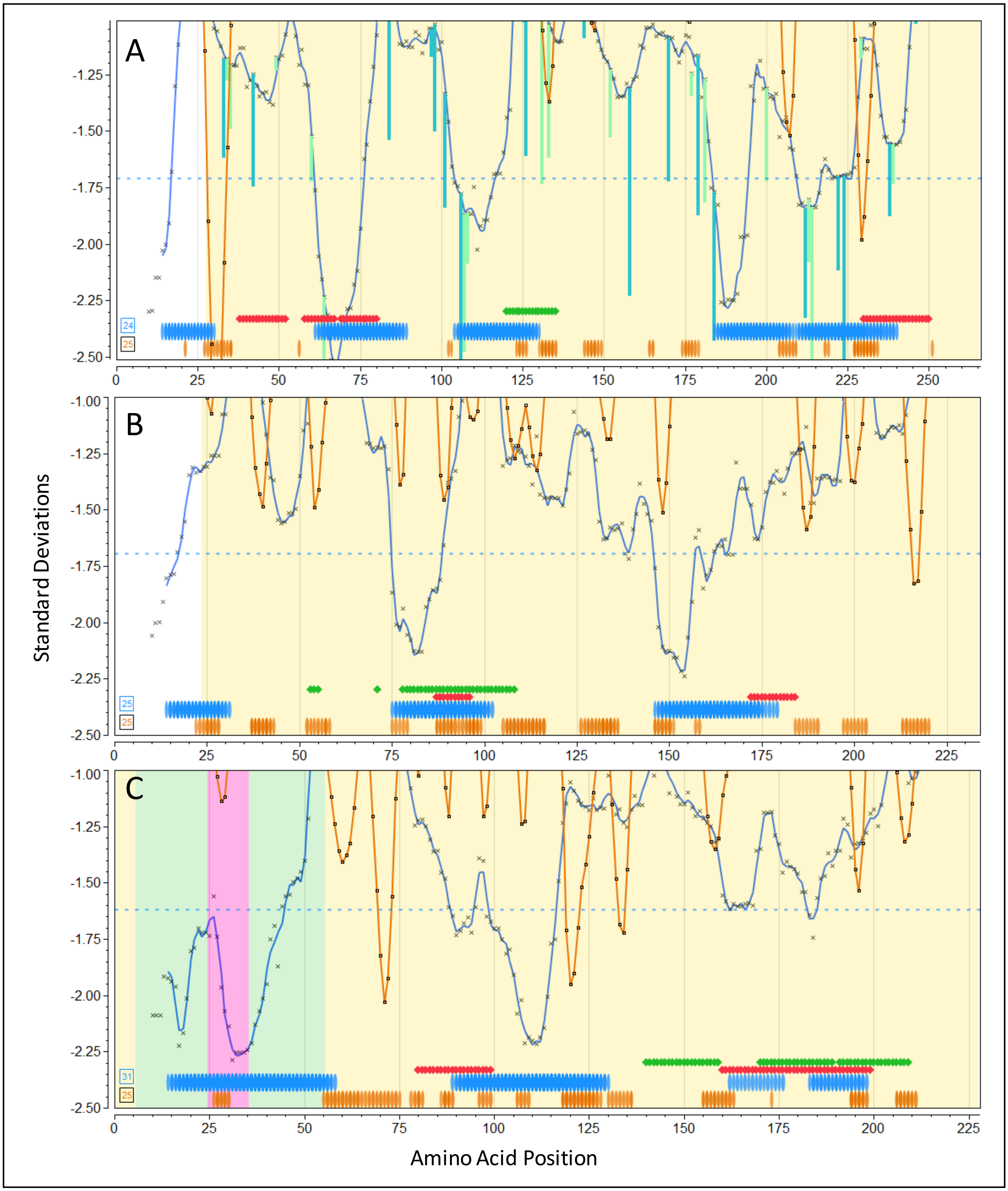

Overlay epitope maps of three toxins from *Staphylococcus aureus*.

Symbols as described in Figure 5; experimental mapping as described in Table 7. (A) Staphylococcal enterotoxin B (GI:57651597). The colored vertical lines are the positions of the N-terminus of high affinity 15-mer binding for DR3 and the linked DRB1*0301 alleles used by Rajagopolan [1] (B) Toxic shock syndrome Toxin 1 (GI:82750121); (C) Thermonuclease precursor (GI:57650135).

***Staph. aureus* Enterotoxin B (SEB) NC_002951.57651597**

Staph. aureus produces a number of toxins that have been studied extensively and we show the overlay maps of three examples. These molecules are important virulence factors but are not strain conserved and several different forms occur in any one strain. Various coincidences between predicted and experimental data are shown in Figure A, B, and C, but any conclusions are tenuous, as the experimental results are consolidated from studies mapping B-cell epitopes in rabbits, humans and mice and MHC binding data is exclusively obtained from the use of mouse models. A recent study examined the immunological response to SEB in transgenic mice carrying the human DRB3 MHC-II [1]. The vertical blue lines in Figure A show the peptides predicted to bind with high affinity to DRB3*0101 and its genetically linked DRB1*0301.

Additional Figure S3d. *Staph aureus* Protein A

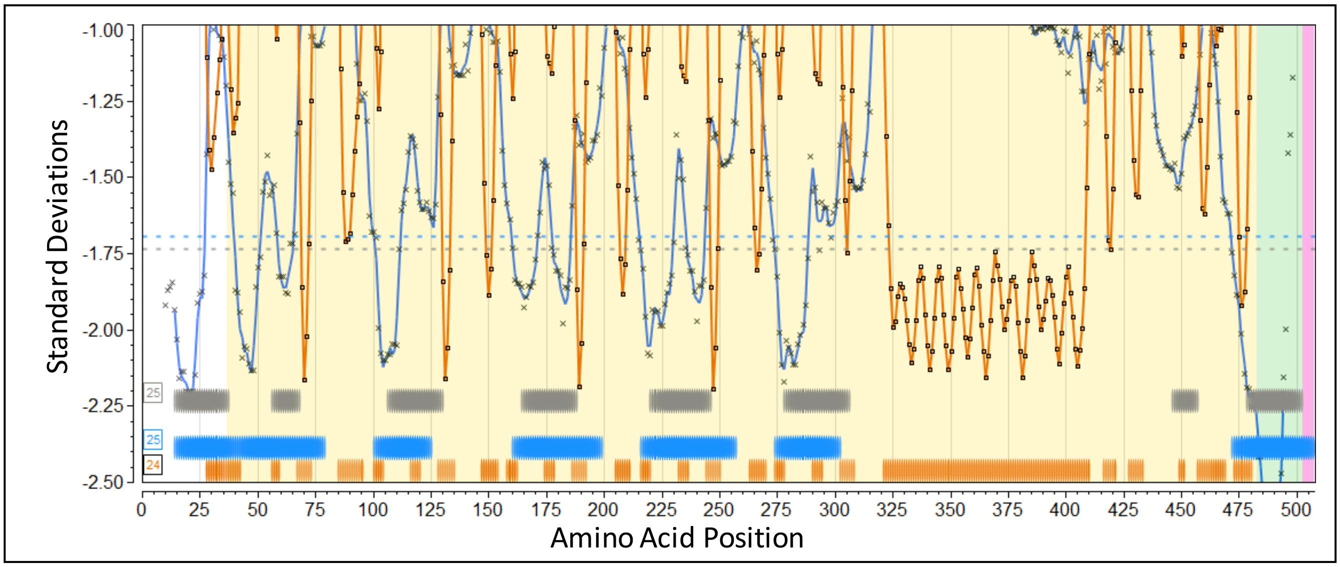

Epitope map of IgG binding protein A from *Staphylococcus aureus* COL (GI 57652458).

Lines and symbols as described in Figure 5. Gray bars depicting MHC-I epitope regions demonstrate the overlapping epitope space sampled by both MHC-I and MHC-II. Also shown is an extended region lacking any MHC binding but with high probability of B-cell epitopes.

Reference List

1. Rajagopalan G, Tilahun AY, Asmann YW, David CS: **Early gene expression changes induced by the bacterial superantigen staphylococcal enterotoxin B and its modulation by a proteasome inhibitor**. *Physiol Genomics* 2009, **37:**279-293.
