# Supplementary material for: An integrated approach to epitope analysis II: A system for proteomic-scale prediction of immunological characteristics
Source: Immunome Res. 2010 Nov 2;6:8. doi: 10.1186/1745-7580-6-8 (PMC2991286; doi:10.1186/1745-7580-6-8)
Supplement: Additional File 4 — Process description (PDF). [file 1745-7580-6-8-S4.PDF]

Additional File 4: Detailed Process Description

The following provides a more detailed description of the considerations in design of an integrated epitope analysis system and should be read in reference to Figure 1.

Process A: Establishment of Parameters for Assessment of MHC binding

In a prior paper (Bremel and Homan; *submitted herewith*) we discussed the rationale for the components of Process A. Principal component amino acid analysis was carried out on the physical properties of amino acids measured in a total of thirty-one different published studies. The first three principal components (also sometimes called "principal properties") account for 89.2% of the total variance. Each amino acid is assigned 3 numerical values based its principal components. This type of descriptor is commonly used in QSAR analysis where it is known as the "z"-scale [1, 2]. It should be noted that this is unrelated to the undefined “Z” scale terminology adopted by DeGroot et al in the EpiMatrix program [3]. Principal components are a pillar of the field of chemometrics where the relationship to binding site energetics is well understood. Several other recent publications have explored approaches to QSAR in MHC binding [4, 5]. While most immunoinformatics has been based on alphabetic sequences coupled to substitution matrices, our approach uses a neural network regression approach. In this approach, for MHC-II binding, each amino acid in a 15-mer is replaced by three z-scale descriptors. $\{z_1(aa_1),z_2(aa_1),z_3(aa_1)\}$, $\{z_1(aa_2),z_2(aa_2),z_3(aa_2)\}$ ...$\{z_1(aa_{15}),z_2(aa_{15}),z_3(aa_{15})\}$. A 9-mer for MHC-I analysis has a correspondingly smaller set of descriptors. These descriptors are uncorrelated, mutually orthogonal metrics that embody about 90% of the variance in all physical properties of the 20 amino acids commonly found in proteins (post-translational modifications can be added but have not been used in our model). The z-scales are thus not in themselves physical properties, but rather uncorrelated dimensionless proxies for amino acid physical properties that can be used predictively: z_1 is a hydrophobicity or polarity correlate, z_2 a size correlate and z_3 an electronic correlate. A characteristic of principal component analysis is that it also produces a set of descriptors that are appropriately weighted for regression analysis. Appropriate weighting is critical in regression predictions generally and a characteristic difficult to attain and assess in substitution-matrix approaches. Use of the z-scales in a neural net regression system, along with a training set of peptides with known ic_{50} (the concentration at which half the peptide can be displaced from the binding site), produces a series of equations that predict the binding affinity of an MHC molecule to a high degree of accuracy and for any peptide for which the NN has been trained. In the accompanying paper we compare our predictions to those of other existing methods.

The primary advantage of this process is that a supercomputer is not required to conduct proteomic scale work. The time consuming process is the NN training, but once the set of prediction equations have been developed it is possible to compute the affinities for all proteins of interest very quickly in a familiar spreadsheet-like paradigm. After the computations are complete, a series of additional statistical and computational activities produce the overlays for visualization of the underlying immunological information. The work is done with the statistical analysis and data visualization application JMP® (or JMP® Genomics). Below we describe this process in detail and provide examples of

the types of immunological characteristics that can be identified, and some heretofore unreported. The overall process is shown in Figure 1.

The final output of Process "A" (**Step 5** in Figure 1) is a NN trained to predict binding to each different HLA supertype, which produces a set of equations that describe and predict the contribution of the physical properties of each amino acid to $\ln(ic_{50})$. Interestingly, as the ic_{50} is directly related to the k_d the physical properties of the amino acids are being used to predict another physical property, a number directly related to a thermodynamic property the Gibbs free energy: $\Delta G^0 = -RT \ln K$. These equations were stored within JMP® for prediction of binding affinities of other peptides of equivalent length. The JMP® statistical application that was used to produce the NN fits stores equations to define columns of numbers; essentially a macro defining the NN output is connected to a column for each allele prediction.

Each overlapping peptide in the proteome is assigned to one row in the data table. The number of columns in the data table varies depending on the size of peptide and the number of MHC supertype affinities being predicted. Using the methodology above, predictive NN were developed for 35 MHC-I and 14 MHC-II molecules. The NN prediction equations were stored in the JMP® platform system so that they could be applied to peptides from various proteomes (Process B).

Process "B": Determination of predicted peptide binding to MHC-I and MHC-II

The goal of this process is to take the entire proteome of an organism (Figure 1, Step 1 Proteome input) and create a series of N-mers for the proteome where each N-mer is offset +1 in the protein starting from the proteins' N-terminus (123456…, 234567… etc.) (Step 6). Then, each amino acid in each peptide is converted to be represented as 3 numbers based on the principal components (Step 7) stored as result of Process A. Thus, each 9-mer in the proteome is represented as a vector of 27 numbers and each 15-mer as a vector of 45 numbers. Then, by applying the prediction equations derived in Step 5 in Process A on the output of Step 7 the $\ln(K_d)$ is predicted for all MHC supertypes for which training sets were available and that were used to "train" the NN. The results (Step 10) are stored in a database table by Genome.GI.N.C providing a means of precise identification of the locations within any particular genome.

Process "C": Determination of protein topology and of predicted B-cell epitope binding of peptides

Proteomes (Step 1) are submitted to one of several publicly available programs for protein topology predictions (Step 8). We have variously used PHOBIUS [6], PHILIUS [7], MEMSAT [8] and TMH [9]. These programs are quite accurate and are used by genomic database centers as a components in the curation of genomes. The output of these programs is a topology prediction for each amino acid in the protein as being intracellular, extracellular, within a membrane or a signal peptide. It is also possible to obtain the actual Bayesian posterior probabilities from the programs as well, but for our purposes it was not particularly helpful and a simple classification is adequate. The result is a data table with the same number of rows as there are amino acids in the proteome coded as Genome.GI.N.topology coded as indicated.

In Step 9 a determination of B-cell epitope predictions is made. Unlike the MHC predictions which provide a predicted affinity, in the case of B-cell epitopes we are making a binary "yes-no" probability prediction that a specific amino acid lies in a B-cell epitope. The B-cell epitope probability may be achieved by submission to one of several publicly available programs for B-cell epitope predictions [10]. Despite reservations about the "underperfomance" [11] these programs have accuracies similar to one another, but generally somewhat lower as compared to topology predictions. As the B-cell epitope predictions rely heavily on the physicochemical properties of amino acids we were able to generate a B-cell epitope prediction system based on principal components whose output is indistinguishable from those available on the internet, but which operates as an integral component of the processes we run.

The output of Step 9 in the process is a Bayesian probability for each amino acid in the protein being located in a B-cell epitope and a corresponding data table with the same number of rows as there are amino acids in the proteome coded as Genome.GI.N.bepi_probability.

Process "D": Assembly of B-cell epitope and MHC binding data.

The results of Steps 8, 9 and 10 were placed into a master data table for further analysis (**Step 11**). Each row in the database table contains a peptide 15-mer and each row indexes the peptide by +1 amino acid. For simplicity, the 9-mer used for MHC-I predictions is also used as the "core" peptide with a tripeptide on each end, to comprise the 15-mer used to assess MHC-II binding. The datatables are maintained sorted by Genome, GI within the genome and N-terminus of the 15-mer peptide within GI (i.e. protein sequence). The method used for assembling epitopes from the database is a "self-organizing map" (SOM) algorithm to which various immunologically relevant screening thresholds can be applied.

The array of genetic variants (supertypes) of HLA molecules in the human population exceeds that for which there are peptide training sets. Additionally, and yet further increasing the combinatorial possibilities, is the fact that each individual has both parental genotypes of MHC on their cell membranes. Despite the combinatorial complexity, examination of the statistics of the predicted binding affinities to a number of different proteins in the proteome of *Staphylococcus aureus* gave rise to several discoveries which suggested that it would be possible to derive a system for determining the probability of binding not only for single supertypes, but for all combinatorial supertypes for which a trained NN was available. The approaches outlined above make it possible to put entire proteomes (or multiple proteomes) consisting of millions of binding affinities into a single data table, in a familiar spreadsheet interface on a standard personal workstation computer (high end better, obviously).

An affinity (defined experimentally as an ic_{50}, the concentration at which half the peptide can be displaced from the binding site) of 500 nM has been widely used to define a "weak binder" (WB) in immunoinformatics prediction schemes [12]. We note that the results obtained for MHC-II binding with the *Staph aureus* COL surfome, the average peptide is classified in the weak binder range. A "strong binder" (SB) is deemed to have a dissociation constant of less than 50 nM. For the *Staph aureus* surfome the SB threshold lies somewhere between the mean minus 1 standard deviation (80.2 nM) and the 10 percentile point (44.7 nM). Since the 10 percentile was quite close to 50 nM point commonly used to conceptualize a strong binder and it is a standard useful statistical cutoff, we found the 10 percentile point as a useful threshold to derive the combinatorial statistics for the various MHC-II supertypes. It is

obvious that other thresholds can be used that will give somewhat different results. Further, as other training sets become available (e.g. for DQB and DPA) it should be possible to extrapolate the general molecular concepts and add these.

Supplemental Table 3b provides the detailed binding affinity for heterozygous and homozygous pairs derived from the training sets.

Supplemental File 3 Shows the output of standardization of surfome proteins for *Staph aureus*

Permuted MHC binding affinities

The equation for calculating the genotypic permutations to effectively provide a population phenotype is:

$$S = \frac{P_r^n}{2} + n$$

Where

S = diploid genetic combinations of MHC alleles

$P = \frac{n!}{(n-r)!}$ permutations of some relevant metric

$n =$ number of MHC alleles

For 14 human MHC-II alleles S= 105 and for 35 MHC-I alleles S = 630.

Process "E": Determination of epitopes conserved across organismal strains

One of the issues that arises when dealing with multiple proteomes is the question of determining which proteins are realistic matches to proteins in another strain of the same organism. Genbank does not make this linkage, nor provide a means to do so. This determination could be a very computationally intensive operation (all vs. all). We devised a practical system to assign proteins into sets based on their size and amino acid sequence across different organismal strains. Various methods could be used to accomplish this; for example, multiple alignment procedures such as BLAST could be used. We found that by re-coding the amino acid sequence into a vector consisting of the 1st principal component of the particular amino acid, i.e. as a number (~polarity score) rather than an alphabetic character, we were able to group proteins which were within ± 20 amino acids in total length and compute a pairwise Pearson correlation coefficient. This detected whether more than one group of proteins was in the set. This proved to be a rapid means of clustering groups of proteins from different strains whose sequences were nearly identical. We found that a pairwise correlation coefficient of 0.8 was a reliable means of grouping proteins. We designated these matched groups as Nearly Identical Protein Sets (NIPS).

Process Output: Epitope Reports

Using standard database management software, output from the various process steps are consolidated into database tables (Step 13). A variety of standard methods and software tools are available for manipulation, extraction, querying, and analysis of data stored in databases. By using standardized

database designs these tools can readily be used individually or in combinations in a variety of reports (Step 14).

Reference List

1. Cruciani G, Baroni M, Carosati E, Clementi M, Valigi R, Clementi S: **Peptide studies by means of principal properties of amino acids derived from MIF descriptors**. *Journal of Chemometrics* 2004, **18:**146-155.

2. Eriksson L, Johansson E, Kettaneh-Wold N, Trygg J, Wikstrom C, Wold S: *Multi and Megavariate Data Analysis.Part I: Basic Principles and Applications*. 2nd edition: Umetrics Academy, Umea, Sweden; 2006.

3. De Groot AS, Martin W: **Reducing risk, improving outcomes: bioengineering less immunogenic protein therapeutics**. *Clin Immunol* 2009, **131:**189-201.

4. Doytchinova IA, Walshe V, Borrow P, Flower DR: **Towards the chemometric dissection of peptide--HLA-A*0201 binding affinity: comparison of local and global QSAR models**. *J Comput Aided Mol Des* 2005, **19:**203-212.

5. Zhao C, Zhang H, Luan F, Zhang R, Liu M, Hu Z, Fan B: **QSAR method for prediction of protein-peptide binding affinity: application to MHC class I molecule HLA-A*0201**. *J Mol Graph Model* 2007, **26:**246-254.

6. Kall L, Krogh A, Sonnhammer EL: **A combined transmembrane topology and signal peptide prediction method**. *J Mol Biol* 2004, **338:**1027-1036.

7. Reynolds SM, Kall L, Riffle ME, Bilmes JA, Noble WS: **Transmembrane topology and signal peptide prediction using dynamic bayesian networks**. *PLoS Comput Biol* 2008, **4:**e1000213.

8. Jones DT: **Improving the accuracy of transmembrane protein topology prediction using evolutionary information**. *Bioinformatics* 2007, **23:**538-544.

9. Krogh A, Larsson B, von HG, Sonnhammer EL: **Predicting transmembrane protein topology with a hidden Markov model: application to complete genomes**. *J Mol Biol* 2001, **305:**567-580.

10. Larsen JE, Lund O, Nielsen M: **Improved method for predicting linear B-cell epitopes**. *Immunome Res* 2006, **2:**2.

11. Blythe MJ, Flower DR: **Benchmarking B cell epitope prediction: underperformance of existing methods**. *Protein Sci* 2005, **14:**246-248.

12. Lund O, Nielsen M, Lundegaard C, Kesmir C, Brunak S: *Immunological Bioinformatics*. Cambridge, MA: MIT Press; 2005.
