# Supplementary material for: An integrated approach to epitope analysis II: A system for proteomic-scale prediction of immunological characteristics
Source: Immunome Res. 2010 Nov 2;6:8. doi: 10.1186/1745-7580-6-8 (PMC2991286; doi:10.1186/1745-7580-6-8)
Supplement: Additional File 5 — Global standardization criteria (PDF). [file 1745-7580-6-8-S5.PDF]

Additional File 5: Derivation of the statistical criteria used for binding threshold selections from a global standardization.

Using the standardized (normally distributed with a mean of zero and unit variance) LN(ic50) metrics for allelic pairs of 15-mers and 9-mers the minimum value for the pair was computed within a window ±4 from each position within the protein sequence. A least-squares mean was calculated over all permuted pairs to arrive at a number for each position in the protein sequence. Statistics for these numbers were then computed for a sub-proteome consisting of the membrane proteins and secreted proteins of *Staphylococcus aureus* COL (302,178 peptides). The numbers given are in standard deviation units of the initial standardized population. Both sub-distributions were normally distributed as was the parent distribution. A threshold for any quantile can be computed from these values. For example the 25%-tile = mean -0.67*std dev. A reverse transform of these units can be made to convert the numbers to the experimental ic50 values.

	Alleles	Permuted pairs	Mean	Std Dev
**MHC-II**	14	105	-1.39	0.46
**MHC-I**	35	630	-1.62	0.22
