# Supplementary material for: An integrated approach to epitope analysis II: A system for proteomic-scale prediction of immunological characteristics
Source: Immunome Res. 2010 Nov 2;6:8. doi: 10.1186/1745-7580-6-8 (PMC2991286; doi:10.1186/1745-7580-6-8)
Supplement: Additional File 6 — Complete dataset summarized in Table 3 (PDF). [file 1745-7580-6-8-S6.PDF]

Additional File 6: Complete Data Set

Ten percentile MHC II binding affinity statistics for 105 different heterozygous and homozygous supertype combinations for 15-mer peptides from the the surface proteome of Staphylococcus aureus COL. The results were obtained using 14 MHC II supertypes for which training sets were available to train the NN. The surface proteome is defined as proteins that are predicted to have one or more transmembrane helices and are therefore expected to be inserted into the cell membrane.

S1	S2	10%tile S1	10%tile S2	10%tile Average	10%tile min of pair
DRB1_0101	DRB1_0101	0.54	0.54	0.54	0.54
DRB1_0301	DRB1_0101	3.81	0.54	2.175	0.54
DRB1_0401	DRB1_0101	1.95	0.54	1.245	0.54
DRB1_0404	DRB1_0101	1.63	0.54	1.085	0.54
DRB1_0405	DRB1_0101	1.92	0.54	1.23	0.54
DRB1_0701	DRB1_0101	0.62	0.54	0.58	0.54
DRB1_0802	DRB1_0101	4.48	0.54	2.51	0.54
DRB1_0901	DRB1_0101	2.64	0.54	1.59	0.54
DRB1_1101	DRB1_0101	2.35	0.54	1.445	0.54
DRB1_1302	DRB1_0101	4.62	0.54	2.58	0.54
DRB1_1501	DRB1_0101	2.31	0.54	1.425	0.54
DRB3_0101	DRB1_0101	5.74	0.54	3.14	0.54
DRB4_0101	DRB1_0101	2.81	0.54	1.675	0.54
DRB5_0101	DRB1_0101	1.58	0.54	1.06	0.54
DRB1_0701	DRB1_0701	0.62	0.62	0.62	0.62
DRB1_0701	DRB1_0405	0.62	1.92	1.27	0.62
DRB1_0802	DRB1_0701	4.48	0.62	2.55	0.62
DRB1_0701	DRB1_0404	0.62	1.63	1.125	0.62
DRB1_0901	DRB1_0701	2.64	0.62	1.63	0.62
DRB1_0701	DRB1_0401	0.62	1.95	1.285	0.62
DRB1_1101	DRB1_0701	2.35	0.62	1.485	0.62
DRB1_0701	DRB1_0301	0.62	3.81	2.215	0.62
DRB1_1302	DRB1_0701	4.62	0.62	2.62	0.62
DRB1_1501	DRB1_0701	2.31	0.62	1.465	0.62
DRB3_0101	DRB1_0701	5.74	0.62	3.18	0.62
DRB4_0101	DRB1_0701	2.81	0.62	1.715	0.62
DRB5_0101	DRB1_0701	1.58	0.62	1.1	0.62
DRB5_0101	DRB5_0101	1.58	1.58	1.58	1.58
DRB5_0101	DRB4_0101	1.58	2.81	2.195	1.58
DRB5_0101	DRB3_0101	1.58	5.74	3.66	1.58
DRB5_0101	DRB1_1501	1.58	2.31	1.945	1.58
DRB5_0101	DRB1_1302	1.58	4.62	3.1	1.58
DRB5_0101	DRB1_1101	1.58	2.35	1.965	1.58
DRB5_0101	DRB1_0901	1.58	2.64	2.11	1.58
DRB5_0101	DRB1_0802	1.58	4.48	3.03	1.58
DRB5_0101	DRB1_0405	1.58	1.92	1.75	1.58

DRB5_0101	DRB1_0404	1.58	1.63	1.605	1.58
DRB5_0101	DRB1_0401	1.58	1.95	1.765	1.58
DRB5_0101	DRB1_0301	1.58	3.81	2.695	1.58
DRB1_0404	DRB1_0404	1.63	1.63	1.63	1.63
DRB1_0404	DRB1_0401	1.63	1.95	1.79	1.63
DRB1_0405	DRB1_0404	1.92	1.63	1.775	1.63
DRB1_0404	DRB1_0301	1.63	3.81	2.72	1.63
DRB1_0802	DRB1_0404	4.48	1.63	3.055	1.63
DRB1_0901	DRB1_0404	2.64	1.63	2.135	1.63
DRB1_1101	DRB1_0404	2.35	1.63	1.99	1.63
DRB1_1302	DRB1_0404	4.62	1.63	3.125	1.63
DRB1_1501	DRB1_0404	2.31	1.63	1.97	1.63
DRB3_0101	DRB1_0404	5.74	1.63	3.685	1.63
DRB4_0101	DRB1_0404	2.81	1.63	2.22	1.63
DRB1_0405	DRB1_0405	1.92	1.92	1.92	1.92
DRB1_0405	DRB1_0401	1.92	1.95	1.935	1.92
DRB1_0802	DRB1_0405	4.48	1.92	3.2	1.92
DRB1_0405	DRB1_0301	1.92	3.81	2.865	1.92
DRB1_0901	DRB1_0405	2.64	1.92	2.28	1.92
DRB1_1101	DRB1_0405	2.35	1.92	2.135	1.92
DRB1_1302	DRB1_0405	4.62	1.92	3.27	1.92
DRB1_1501	DRB1_0405	2.31	1.92	2.115	1.92
DRB3_0101	DRB1_0405	5.74	1.92	3.83	1.92
DRB4_0101	DRB1_0405	2.81	1.92	2.365	1.92
DRB1_0401	DRB1_0401	1.95	1.95	1.95	1.95
DRB1_0401	DRB1_0301	1.95	3.81	2.88	1.95
DRB1_0802	DRB1_0401	4.48	1.95	3.215	1.95
DRB1_0901	DRB1_0401	2.64	1.95	2.295	1.95
DRB1_1101	DRB1_0401	2.35	1.95	2.15	1.95
DRB1_1302	DRB1_0401	4.62	1.95	3.285	1.95
DRB1_1501	DRB1_0401	2.31	1.95	2.13	1.95
DRB3_0101	DRB1_0401	5.74	1.95	3.845	1.95
DRB4_0101	DRB1_0401	2.81	1.95	2.38	1.95
DRB1_1501	DRB1_1501	2.31	2.31	2.31	2.31
DRB1_1501	DRB1_1302	2.31	4.62	3.465	2.31
DRB3_0101	DRB1_1501	5.74	2.31	4.025	2.31
DRB1_1501	DRB1_1101	2.31	2.35	2.33	2.31
DRB4_0101	DRB1_1501	2.81	2.31	2.56	2.31
DRB1_1501	DRB1_0901	2.31	2.64	2.475	2.31
DRB1_1501	DRB1_0802	2.31	4.48	3.395	2.31
DRB1_1501	DRB1_0301	2.31	3.81	3.06	2.31
DRB1_1101	DRB1_1101	2.35	2.35	2.35	2.35
DRB1_1101	DRB1_0901	2.35	2.64	2.495	2.35
DRB1_1302	DRB1_1101	4.62	2.35	3.485	2.35
DRB1_1101	DRB1_0802	2.35	4.48	3.415	2.35
DRB3_0101	DRB1_1101	5.74	2.35	4.045	2.35
DRB4_0101	DRB1_1101	2.81	2.35	2.58	2.35
DRB1_1101	DRB1_0301	2.35	3.81	3.08	2.35

DRB1_0901	DRB1_0901	2.64	2.64	2.64	2.64
DRB1_0901	DRB1_0802	2.64	4.48	3.56	2.64
DRB1_1302	DRB1_0901	4.62	2.64	3.63	2.64
DRB3_0101	DRB1_0901	5.74	2.64	4.19	2.64
DRB4_0101	DRB1_0901	2.81	2.64	2.725	2.64
DRB1_0901	DRB1_0301	2.64	3.81	3.225	2.64
DRB4_0101	DRB4_0101	2.81	2.81	2.81	2.81
DRB4_0101	DRB3_0101	2.81	5.74	4.275	2.81
DRB4_0101	DRB1_1302	2.81	4.62	3.715	2.81
DRB4_0101	DRB1_0802	2.81	4.48	3.645	2.81
DRB4_0101	DRB1_0301	2.81	3.81	3.31	2.81
DRB1_0301	DRB1_0301	3.81	3.81	3.81	3.81
DRB1_0802	DRB1_0301	4.48	3.81	4.145	3.81
DRB1_1302	DRB1_0301	4.62	3.81	4.215	3.81
DRB3_0101	DRB1_0301	5.74	3.81	4.775	3.81
DRB1_0802	DRB1_0802	4.48	4.48	4.48	4.48
DRB1_1302	DRB1_0802	4.62	4.48	4.55	4.48
DRB3_0101	DRB1_0802	5.74	4.48	5.11	4.48
DRB1_1302	DRB1_1302	4.62	4.62	4.62	4.62
DRB3_0101	DRB1_1302	5.74	4.62	5.18	4.62
DRB3_0101	DRB3_0101	5.74	5.74	5.74	5.74
	**Mean**	**2.92**	**2.37**	**2.64**	**1.88**
	**Std Dev**	**1.47**	**1.41**	**1.07**	**1.08**
