# Supplementary material for: An integrated approach to epitope analysis II: A system for proteomic-scale prediction of immunological characteristics
Source: Immunome Res. 2010 Nov 2;6:8. doi: 10.1186/1745-7580-6-8 (PMC2991286; doi:10.1186/1745-7580-6-8)
Supplement: Additional File 7 — Vaccinia additional figures (PDF). [file 1745-7580-6-8-S7.PDF]

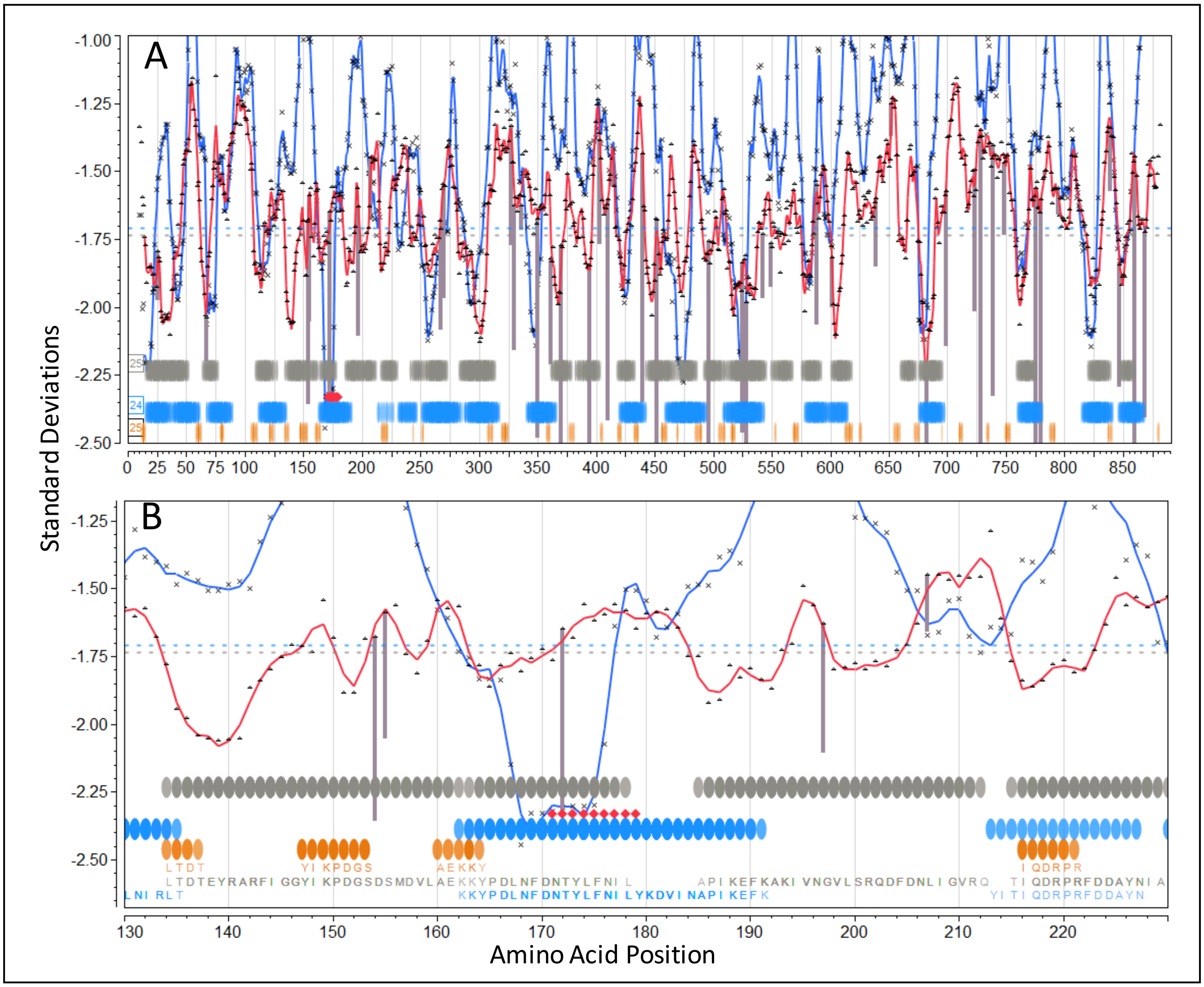

Overlay epitope maps of locus A10L (GI:68275926) from Vaccinia virus Western Reserve.

Overlay is shown at two different resolutions showing MHC-I 9-mer peptides mapped in HLA A*1101/K^b transgenic mice[1]. Symbols as described in Figure 5. Vertical lines are the N-terminal positions of predicted high affinity binding 9-mer peptides for B*1101 predicted by neural net regression. Background is unshaded because this protein is predicted to lack any membrane domains.

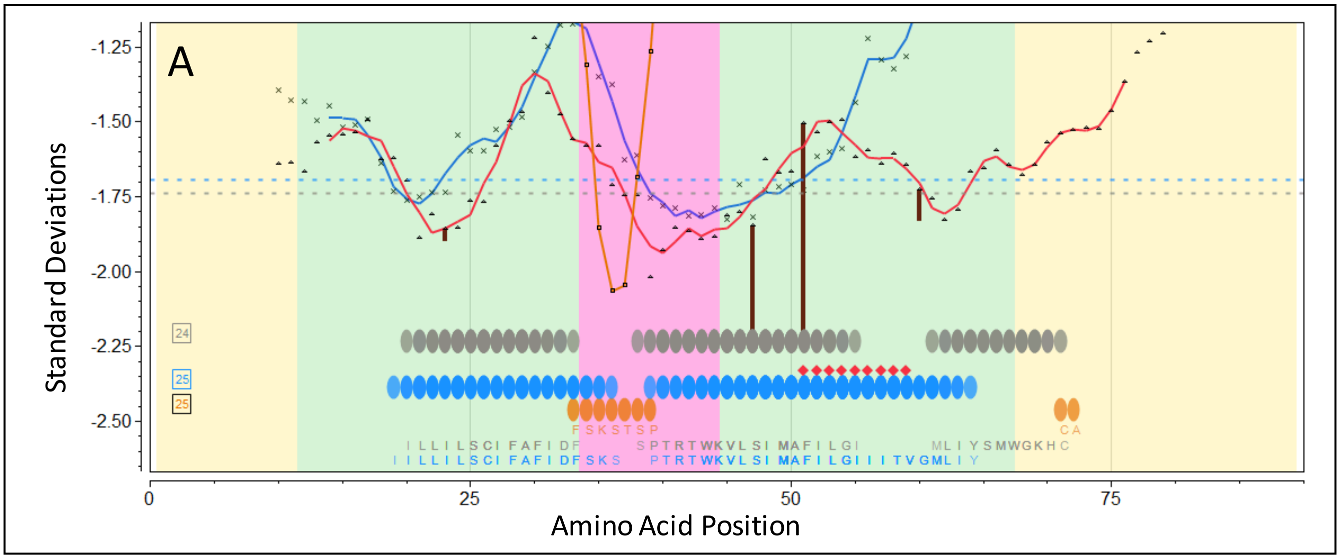

Overlay epitope maps of locus A14L (GI:68275930) from Vaccinia virus Western Reserve.

Overlay is shown at two different resolutions showing MHC-I 9-mer peptides mapped in HLA A*0201/K^b transgenic mice[1]. Symbols as described in legend to Figure 6. Vertical lines are the N-terminal positions of predicted high affinity binding 9-mer peptides for A*0201 predicted by neural net regression.

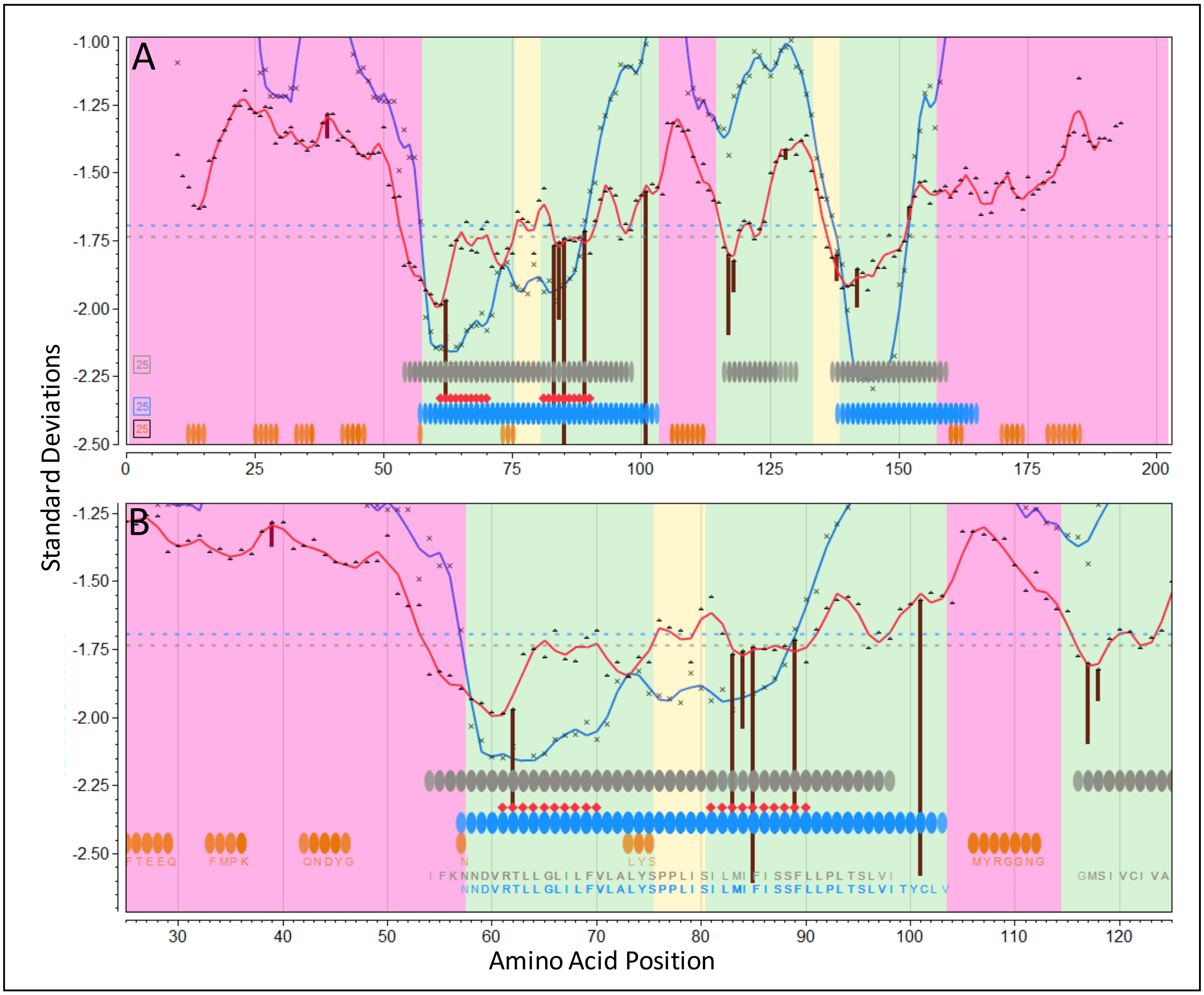

Overlay epitope maps of locus A17L (GI:68275934) from Vaccinia virus Western Reserve.

Overlay is shown at two different resolutions for MHC-I 9-mer peptides mapped in HLA A*0201/K^b transgenic mice [1]. Symbols as described in Figure 5. Vertical lines are the N-terminal positions of predicted high affinity binding 9-mer peptides for A*0201 predicted by neural net regression.

The experimentally mapped epitopes are found to be entirely within predicted transmembrane domains. The blue and gray bands through the regions indicate that both predicted MHC-I and MHC-II peptides in the region are predicted to have a high affinity. The coincidence of these bands also supports the concept that the two classes of MHC apparently sample a common type of epitope structural space.

Reference List

1. Pasquetto V, Bui HH, Giannino R, Banh C, Mirza F, Sidney J, Oseroff C, Tscharke DC, Irvine K, Bennink JR, Peters B, Southwood S, Cerundolo V, Grey H, Yewdell JW, Sette A: **HLA-A*0201, HLA-A*1101, and HLA-B*0702 transgenic mice recognize numerous poxvirus determinants from a wide variety of viral gene products**. *J Immunol* 2005, **175:**5504-5515.
